# Effect of different types of biochar on soil properties and functional microbial communities in rhizosphere and bulk soils and their relationship with CH_4_ and N_2_O emissions

**DOI:** 10.3389/fmicb.2023.1292959

**Published:** 2023-11-02

**Authors:** Jian-Qing Qi, Hai-Yan Yuan, Qi-Lu Zhuang, Eric-Fru Zama, Xiao-Fei Tian, Bao-Xian Tao, Bao-Hua Zhang

**Affiliations:** ^1^School of Geography and Environment, Liaocheng University, Liaocheng, China; ^2^Department of Agricultural and Environmental Engineering, College of Technology, University of Bamenda, Bambili, Cameroon

**Keywords:** biochar, rhizosphere, bulk, CH_4_, N_2_O, *pmoA*, *nosZ*

## Abstract

Biochar as an agricultural soil amendment plays vital roles in mediating methane (CH_4_) and nitrous oxide (N_2_O) emissions in soils. The link between different types of biochar, bulk soil, and rhizosphere microbial communities in relation to CH_4_ and N_2_O emissions is being investigated in this study. The rice pot experiment was conducted using biochar at two temperatures (300°C and 500°C) in combination with three biochar levels (0, 2, 10% w/w). Soil properties and the abundance of genes associated with CH_4_ and N_2_O emissions from both rhizosphere and bulk soils were investigated. The study also aimed to examine the structure of microbial communities (*pmoA*, *nosZ*) in rhizosphere and bulk soils whereas CH_4_ and N_2_O emissions were monitored while growing rice. Results showed that biochar at 300°C and 10% incorporation significantly increased the CH_4_ emissions by up to 59% rise compared to the control group. Random Forest analysis revealed that the ratio of *mcrA/pmoA* along with the abundance of *mcrA* from both rhizosphere and bulk soils, the abundance of *AOA*, TN, DOC, and the community composition of *pmoA*-harboring microorganisms from both bulk and rhizosphere soils were important predictors of CH_4_ emissions. Therefore, the ratio of *mcrA/pmoA* in rhizosphere soil and the abundance of *AOA* in bulk soil were the main factors influencing CH_4_ emissions. Variation Partitioning Analysis (VPA) results indicated that the effects of these factors on bulk soil were 9% of CH_4_ emissions variations in different treatments, which contributed more than rhizosphere soils’ factors. Moreover, random forest analysis results indicated that the abundance of *AOB* in bulk soil was the most important predictor influencing N_2_O emissions. The VPA result revealed that the factors in rhizosphere soil could explain more than 28% of the variations in N_2_O emissions. Our study highlights that rhizosphere soil has a more significant effect than bulk soil on N_2_O production. Our findings further the understanding of the link between bulk and rhizosphere attributes, and their impact on CH_4_ and N_2_O emissions in paddy soils. In summary, we recommend the application of biochar at 500°C and 2% incorporation rate for agricultural production in the area.

## 1. Introduction

Greenhouse gases present a significant contemporary environmental concern. The relentless increase in greenhouse gas emissions (Smith and Fang, 2010), is expected to have far-reaching impacts on both human development and the stability of natural ecosystems, primarily through the phenomenon of global warming. Among the greenhouse gases, including carbon dioxide (CO_2_), nitrous oxide (N_2_O) and methane (CH_4_), CO_2_ is the most widespread while CH_4_ and N_2_O are the most potent with global warming potentials of 298 and 23 times that of CO_2_ (Munoz et al., 2010). Agricultural land has been identified as a major source of CH_4_ and N_2_O. Rice cultivation has contributed up to 20% of all CH_4_ and N_2_O emissions (FAO, 2020), with average annual emissions of 88.0–98.1 Gg and 7.22–8.64 Tg, respectively (Liu X. et al., 2019). Given that rice serves as the main staple cereals, constituting a substantial 25.8% of the national total sown crop area in China (Wu et al., 2023), mitigating N_2_O and CH_4_ emissions in paddy soils is crucial as global warming worsens.

Biochar is an inert carbon-rich product generated from the restricted oxygen pyrolysis of biomass residues (Chen W. et al., 2019). Biochar has high alkalinity, rich carbon content, abundant porous structure, and a high surface area which is determined by pyrolysis temperatures (Khaledi et al., 2023). It has been widely utilized for enhancing soil viability and promoting crop growth (He et al., 2017; Xu et al., 2022). In addition, some studies reported that the utilization of biochar as a soil amendment could decrease CH_4_ and N_2_O emissions from paddy soils (Wang et al., 2018). Contrarily, some studies showed that biochar increased CH_4_ and N_2_O emissions while others demonstrated no significant effect (Cheng et al., 2012; Troy et al., 2013). Taking the aforementioned studies into account, there is inconsistent information regarding the effect of biochar on CH_4_ and N_2_O emissions and their possible relation to the basic properties of biochar. The pyrolysis temperature of biochar is what determines its properties. Prior studies have found that biochar amendments, particularly those produced at specific pyrolysis temperature of 500°C and 700°C, have been associated with reduced N_2_O emissions and increased CH_4_ emissions (Wang et al., 2017). Meta-analysis results have shown that change in pyrolysis temperature does not result in significant differences in N_2_O emissions; however, pyrolysis temperature below 600°C significantly increase CH_4_ emissions (Shakoor et al., 2021). The effect of biochar on CH_4_ and N_2_O emissions also varies with different biochar incorporation rates. Prior research has demonstrated that biochar application rates of 10 (t ha^−1^), 20 (t ha^−1^), and >40 (t ha^−1^) lead to significant increases in N_2_O emissions. Further, at rates of 20 and 40 (t ha^−1^), there is a concurrent rise in CH_4_ emissions (Shakoor et al., 2021). Nevertheless, Tarin et al. (2021) reported that with the increase of biochar incorporation rates, the mitigation effect on soil CH_4_ emission became more obvious. Moreover, previous studies have focused on either the effects of different temperatures, or different biochar incorporation rates on CH_4_ and N_2_O emissions; however, few studies concurrently examined the combined effects of different temperatures and various biochar incorporation rates on CH_4_ and N_2_O emissions.

N_2_O and CH_4_ emissions from paddy soils involve complex biological processes. Microorganisms are the key mediators of CH_4_ and N_2_O emissions from the soil and play crucial roles in CH_4_ and N_2_O production and consumption processes. The addition of biochar offers adequate habitat for microorganisms, thereby enhancing the diversity of soil microbial populations (Liu X. R. et al., 2019). Currently, the study of microorganisms in rhizosphere soils has gained much interest within the realm of scientific research. Rhizosphere soil constitutes the soil region adjacent to plant roots, serving not only as a habitat for soil microorganisms but also as a key factor that influences soil CH_4_ and N_2_O emissions (Zhong et al., 2022). Previous research has shown that rhizosphere microbial communities are distinct from those found in bulk soil (Li et al., 2019). The functional community of rhizosphere microorganisms is influenced by the interactions between these microbes and plants. Plant root exudates have the remarkable capacity to substantially boost microbial activities within the rhizosphere (Zhou et al., 2018; Ibrahim et al., 2020). Previous research showed that rhizosphere soil had a greater effect on CH_4_ and N_2_O emissions than bulk soil (Li S. et al., 2022; Zhong et al., 2022). However, majority of studies have concentrated on examining the effects of different types or amounts of biochar amendments on the bulk soil microbial communities (Wang et al., 2017, 2023; Phillips et al., 2022). Only a limited number of studies have delved into the impacts of the interaction of different types and amounts of biochar amendments on the microbial communities within both rhizosphere and bulk soils, as well as the link between them and greenhouse gas emissions. As the rhizosphere zone serves as an important interface for root-soil-microorganism interactions, it is crucial to examine the impact of both biochar addition on rhizosphere microbial functional communities, and any possible links between these communities, the soil attributes, CH_4_ and N_2_O emissions.

Therefore, this pot study aimed to achieve three primary objectives: (1) determine the abundance of relevant functional microorganisms involved in N_2_O and CH_4_ production and consumption in rhizosphere and bulk soils using quantitative polymerase chain reaction (qPCR) assays; (2) examine the community structures of methanotrophs using the *pmoA* gene, and evaluate denitrifying bacterial communities by the *nosZ* gene in rhizosphere and bulk soils by performing sequencing analysis on an Illumina MiSeq platform; and (3) explore the relationship between soil abiotic and biotic factors in both bulk and rhizosphere soils, the CH_4_ and N_2_O emissions from paddy soils with different temperatures, and the different incorporation rates of biochar amendment.

## 2. Materials and methods

### 2.1. Soil and biochar

Paddy soil sampling site was located at Yutai Village, Dong’e County, Liaocheng City, Shandong Province, China (116°12′ E, 36°11′ N). This area is classified as fluvo-aquic. This area experiences warm temperate continental monsoon climate, with an average temperature of 13.6°C and 707 mm in annual precipitation. The paddy soil (0–20 cm) was taken from the area where rice was grown for more than 30 years. After air drying, the soil underwent a 2 mm sieving process and any stones or plant residues were removed. The soil physicochemical properties are shown in Supplementary Table S1.

Biochar was produced from rice straw in this experiment. The rice straw was chosen as raw material due to its extensive application within agroecosystems (Park et al., 2019; Xu et al., 2019). The rice straw was first air-dried, cut into small segments, fed into the pyrolyzing furnace, and closed tightly. The rice straw was pyrolyzed under hypoxic conditions with a slow-pyrolysis process. Pyrolyzing temperature was increased to either 300°C or 500°C at a rate of 8.5°C min^−1^ and maintained for approximately 8 h. The total nitrogen (TN) and total carbon (TC) content of biochar pyrolyzed at 300°C was 17.7 g kg^−1^ and 523 g kg^−1^, respectively. TN and TC content of biochar pyrolyzed at 500°C was 582.6 g kg^−1^ and 12.4 g kg^−1^.

### 2.2. Pot experimental setup

The pot experiments, which began during the paddy season in June 2021, were conducted in Liaocheng University, Shandong Province, China. Each pot contained 5 kg of soil, with 1 kg of soil placed inside a root bag designated as rhizosphere soil, while the remaining 4 kg of soil situated outside the root bag was categorized as bulk soil. We employed the traditional and high-quality cultivar “Huang-hua-zhan,” sourced from the Rice Research Institute, Guangdong Academy of Agricultural Sciences, China, for our potting experiments. Two biochar levels (2 and 10%) and two pyrolysis temperatures (300°C and 500°C) were used in the experiment. The experiment was set up with five treatments: (1) CK, no biochar addition (control); (2) B300-M, biochar at 300°C and 2% (w:w) addition; (3) B300-H, biochar at 300°C and 10% (w:w) addition; (4) B500-M, biochar at 500°C and 2% (w:w) addition; (5) B500-H, biochar at 500°C and 10% (w:w) addition. 2% (w:w) biochar was chosen because it represents a common field amendment rate of 24 t ha^−1^, while 10% (w:w) biochar was chosen to exaggerate biochar influence on soil biogeochemistry (Harter et al., 2014). Moreover, 10% (w:w) is similar to the amounts of biochar found in the archeological black soil of the Amazon in Brazil (Atkinson et al., 2010). The experiment was carried out in triplicate and in each treatment, three identical pots (260 mm × 300 mm) were set up, each containing the required type and amount of biochar along with 5 kg of soil (dry weight). Before being added to the pots, different types and amounts of biochar were mixed with the soil in accordance with their treatments. The prepared pots were subsequently submerged in water for 10 days, and three rice seedlings were then inserted into each pot. The water level in the pots was maintained at 2 cm above soil surface during the rice growth. Nitrogen fertilizer was applied at rate of 200 mg N kg^−1^, urea was added as a basal fertilizer at a ratio of 3:2, and one topdressing was applied in five distinct treatments. Calcium superphosphate (90 mg P_2_O_5_ kg^−1^) and potassium chloride (105 mg K_2_O kg^−1^) were applied uniformly as additional basal fertilizers. Once the rice had matured (i.e., 87 days after transplanting), destructive sampling of the soil from the root bag and outside of the root bag was carried out. The former represented the rhizosphere soils with the representative numbers: R-CK, rhizosphere soils in the CK treatment; R-B300-M, rhizosphere soil in the treatment of B300-M; R-B300-H, rhizosphere soil in the treatment of B300-H; R-B500-M, rhizosphere soil in the treatment of B500-M and R-B500-H, rhizosphere soil in the treatment of B500-H. And the latter refers to bulk soils, which are labeled as B-CK, bulk soil in the CK treatment; B-B300-M, bulk soil in the treatment of B300-M; B-B300-H, bulk soil in the treatment of B300-H; B-B500-M, bulk soil in the treatment of B500-M; and B-B500-H, bulk soil in the treatment of B500-H. Each soil sample was divided into two subsamples. One subsample was air-dried to determine soil physicochemical properties, and the other was stored at −20°C for soil DNA extraction.

### 2.3. CH_4_ and N_2_O measurements and estimation of the GWPt

Soil CH_4_ and N_2_O sampling were consistently carried out throughout the entirety of the rice season, employing the static chamber method following the detailed procedure outlined by Wang et al. (2018). We established an airtight system by affixing a 60 cm tall transparent polyvinyl chloride chamber to the upper rim of the pot. This connection was achieved using a water-filled groove during each gas sampling event. For internal air mixing, a fan was positioned on top of the chamber. Gas samples were taken from each chamber at 0, 30, and 60 min between 5 and 7 p.m. on each sampling day. 50 mL of gas was extracted with a syringe, stored in an air pocket, and transported to the lab for subsequent measurement. An Agilent 7890A gas chromatograph (United States) equipped with an electron capture detector (ECD) and flame ionization detector (FID) was used to determine N_2_O and CH_4_ concentrations. N_2_O and CH_4_ fluxes were calculated by utilizing a linear regression analysis hitch considered the temporal changes in their concentrations within the chamber. Gas fluxes and the cumulative fluxes of N_2_O and CH_4_ were calculated with the equation described by Wang et al. (2018). The cumulative fluxes were determined by aggregating the weekly emissions over the entire duration of the study, following the method outlined by Wang et al. (2018). The global warming potential (GWPt) was calculated with the equation described by Sun et al. (2019).

### 2.4. Soil measurement, DNA extraction, and quantitative PCR assay

The pH levels of both bulk and rhizosphere soils were measured using a pH meter (Wu et al., 2019), with a water-to-soil ratio of 5:1 (v:w) in the soil slurry. The ammonium (NH_4_^+^-N) and nitrate (NO_3_^−^-N) in the bulk and rhizosphere soils were extracted with 0.5 mol L^−1^ KCl and quantified through UV spectrophotometry according to the Indophenol Blue spectrophotometric protocol. Dissolved organic carbon (DOC) levels of the bulk and rhizosphere soils were determined using a total organic carbon (TOC) analyzer (Wang et al., 2021). TC and TN in the bulk and rhizosphere soils were measured with an element analyzer (Wang et al., 2021). The ratio of carbon to nitrogen (C/N) was calculated by dividing bulk and rhizosphere soils TC by their TN concentrations.

For the extraction of DNA from both bulk and rhizosphere soils, as well as subsequent quantitative real-time polymerase chain reaction (qPCR) analyses, we followed the precise methodology outlined by Chetri et al. (2022). A fast DNA Spin kit (MP Biomedicals, Germany) was used to extract 0.5 g of freeze-dried DNA from the bulk or rhizosphere soils. The quality and the quantity of the soil DNA obtained were then verified using a spectrophotometer (Nano Drop Technologies, United States). QPCR was used to quantify the abundance of functional genes involved in the generation and consumption of CH_4_, such as *mcrA* and *pmoA*. It was also used to quantify the production and emission of N_2_O, such as archaeal and bacterial *amoA*, *nirK*, *nirS*, *nosZ*, and *nosZII*. Details about the amplification primer and reaction conditions of the qPCR are described in Supplementary Table S2. The amplification was performed in 25 μL reaction mixtures, consisting of 12.5 μL SYBR green (TaKaRa, Japan), 0.5 μL of forward primer, 0.5 μL of reverse primer, 2 μL of DNA sample, and 9.5 μL of sterilized deionized water. At rice maturity, we collected the rice seeds and straw from each pot to measure the grain yield. The rice seeds and straw were determined after the grains were oven dried at approximately 70°C.

### 2.5. Illumina MiSeq sequencing

Illumina MiSeq sequencing was used to assess the functional microbial community structures, including *pmoA*-harboring and *nosZ*-containing microorganisms in the bulk and rhizosphere soils. The *nosZ* and *pmoA* genes were amplified by the A189f/mb661r (Costello and Lidstrom, 1999) and *nosZ_2R*/*nosZ_2F* (Henry et al., 2006) primer sets, respectively, with a unique 7-bp barcode sequence at the 5′ end of primers. PCR amplification was carried out in a 50 μL reaction volume with 25 μL SYBR Premix Ex Taq™ (TaKaRa, Japan), 1 μL of forward primer, 1 μL of reverse primer, 2 μL of DNA sample, and 21 μL of sterilized deionized water. The reaction conditions are shown in Supplementary Table S2. The PCR products were assessed using agarose gel electrophoresis and purified with the QIAquick PCR purification kit (Qiagen). The obtained purified PCR amplicons were mixed at equimolar ratios, and then sequenced on an Illumina MiSeq PE300 platform in accordance with the manufacturers’ instructions (Shanghai Personalbio Technology Co., Ltd., Shanghai, China). The obtained raw sequencing data were deposited to the Sequence Read Archive (SRA) of NCBI under the accession number PRJNA970957.

The *pmoA*-containing and *nosZ*-harboring microbial raw sequences were processed in accordance with previous protocols (Li C. et al., 2022). Briefly, singletons and low-levels of microbial sequences were removed. The remaining sequences were assigned to operational taxonomic units (OTUs). Illumina MiSeq sequencing yielded a total of 29,824 sequences for the *pmoA* genes and 84,935 sequences for the *nosZ* genes. Taxonomic affiliation of the representative *pmoA* and *nosZ* sequences were assigned with the QIIME2 based on *pmoA* and *nosZ* databases, respectively. To facilitate the comparison of community compositions among *pmoA*-harboring and *nosZ*-containing microorganisms in bulk and rhizosphere soil, the OTU matrices were resampled to a standardized level to ensure uniformity across the different treatments. The alpha-diversity indices including Chao1, Simpson, Shannon, Observed-species, and Pielow_e were calculated based on the resampled OTU table.

### 2.6. Data analysis

R software (4.2.2) and SPSS software (version 19, IMB, Inc., United States) were used to analyze the data obtained in this study. One-way analysis of variance (ANOVA) was utilized to evaluate and determine significant differences in various aspects, including soil physicochemical properties, the abundance of functional microbial gene copies associated with both CH_4_ and N_2_O production and consumption, as well as the composition of *pmoA*-containing and *mcrA*-harboring microbial communities. This analysis was conducted across different treatments, both rhizosphere and bulk soils. Non-metric multidimensional scaling analysis (NMDS) based on the Bray-Curtis distance matrices of communities was performed to analyze *pmoA* and *nosZ* community structures in R using the “vegan” and “ggplot2” packages. Pearson correlation was performed to test the associations between the CH_4_ and N_2_O emission rates, soil properties, and the abundances and community compositions of *pmoA* and *nosZ*. Furthermore, a random-forest machine-learning model was constructed and implemented to assess the relative contributions of various factors in relation to gas emissions. These factors included the physicochemical properties, the abundance of functional genes, and microbial community composition, in bulk and rhizosphere soils. The top 2 components of *pmoA* and *nosZ* microbial compositional variation obtained from NMDS were used as dependent variables in both Pearson correlation and random-forest machine-learning models. Moreover, to explore the microbial interactions in rhizosphere and bulk soils, we constructed species cooccurrence networks at the OTU level. The “igraph” package in R was used to extract the data of the nods and the edges. The network visualization was generated by Gephi.

## 3. Results

### 3.1. Soil properties

The physicochemical properties of both bulk and rhizosphere soils within five distinct treatments, each amended with varying biochar temperatures and incorporation rates, are presented in Table 1. Biochar application increased bulk and rhizosphere soils pH. The pH of the soils changed significantly, ranging from 8.35 to 8.97 in rhizosphere soil, which was higher than that in the bulk soil (8.43–8.59). The NO_3_^−^-N showed the same trend with biochar application in both rhizosphere and bulk soils. The amount of NO_3_^−^-N was higher at 10% biochar addition than that at 2% incorporation regardless of the type of biochar. The fluctuations in NH_4_^+^-N closely mirrored those observed in NO_3_^−^-N levels. Specifically, in both rhizosphere and bulk soils, the biochar at 500°C with a 10% incorporation rate led to a significant increase in NH_4_^+^-N levels, elevating them by 25.94%–37.7%. In comparison to the control group, the addition of biochar resulted in increases in soil DOC with a higher concentration observed in rhizosphere soil. Notable increases in TC, TN, and the C/N ratio were found in both bulk and rhizosphere soils. Particularly, TC and C/N exhibited significant increases. Significant differences emerged when comparing the effects of varying biochar incorporation rates at the same temperature. Specifically, bulk soil had a significantly higher TC content than rhizosphere soil at a 2% biochar incorporation level, while at a 10% incorporation level, the relationship reversed. The rhizosphere soil had a consistently higher TN content than bulk soil except in the case of the treatment at 300°C with a 2% incorporation of biochar. This pattern of C, N content is the opposite of the trend observed for the value of the C/N ratio.

**Table 1:** Soil physicochemical properties in bulk and rhizosphere soils among different treatments with or without biochar amendment.

Treatment	pH	NO_3_^—^N (mg/kg)	NH_4_^+^-N (mg/kg)	DOC (mg/kg)	TC (mg/kg)	TN (mg/kg)	C/N
R-CK	8.35 ± 0.03f	107.50 ± 47.40abc	3.74 ± 0.79 cd	248.17 ± 9.02b	20.50 ± 0.14d	1.07 ± 0.05c	19.25 ± 0.74f
R-B300-M	8.47 ± 0.07def	99.76 ± 38.97abc	3.37 ± 0.55d	380.80 ± 163.87a	28.53 ± 0.25c	1.20 ± 0.08c	23.88 ± 1.59e
R-B300-H	8.97 ± 0.15a	123.57 ± 26.73ab	6.79 ± 0.88 cd	282.82 ± 38.50ab	67.03 ± 5.50a	2.40 ± 0.22a	27.96 ± 0.70c
R-B500-M	8.64 ± 0.05c	62.26 ± 32.12bc	2.99 ± 0.47d	220.71 ± 46.72bc	29.93 ± 1.39c	1.20 ± 0.0001c	24.94 ± 1.16de
R-B500-H	8.83 ± 0.03b	139.64 ± 10.21a	9.70 ± 2.17bc	217.65 ± 52.62bc	66.77 ± 3.36a	2.13 ± 0.26a	31.57 ± 2.35b
B-CK	8.43 ± 0.09ef	95.00 ± 22.02abc	8.13 ± 4.03bcd	103.15 ± 13.18c	21.20 ± 0.65d	1.07 ± 0.12c	20.09 ± 1.83f
B-B300-M	8.49 ± 0.01de	49.17 ± 40.31c	4.04 ± 0.80 cd	112.84 ± 12.45c	29.87 ± 0.82c	1.27 ± 0.05c	23.60 ± 0.66e
B-B300-H	8.59 ± 0.05 cd	139.05 ± 24.15a	13.02 ± 0.02b	163.78 ± 10.72bc	64.23 ± 5.74ab	2.20 ± 0.22a	29.22 ± 0.38bc
B-B500-M	8.55 ± 0.02cde	77.14 ± 44.20abc	4.85 ± 0.11 cd	97.26 ± 1.90c	31.73 ± 0.33c	1.17 ± 0.05c	27.23 ± 0.87 cd
B-B500-H	8.58 ± 0.04 cd	90.83 ± 22.65abc	30.66 ± 7.94a	94.61 ± 7.60c	59.10 ± 2.06b	1.70 ± 0.0002b	34.76 ± 1.21a

The data is represented by their means and standard deviation (*n* = 3). Lowercase letters indicate significant differences among different treatments (*p* < 0.05). CK, without biochar addition; B300, biochar pyrolyzed at 300°C; B500, biochar pyrolyzed at 500°C; M, 2% (w:w) biochar addition; H, 10% (w:w) biochar addition; R, rhizosphere soil; B, bulk soil.

### 3.2. C-related and N-related functional genes

**Figure 1:**
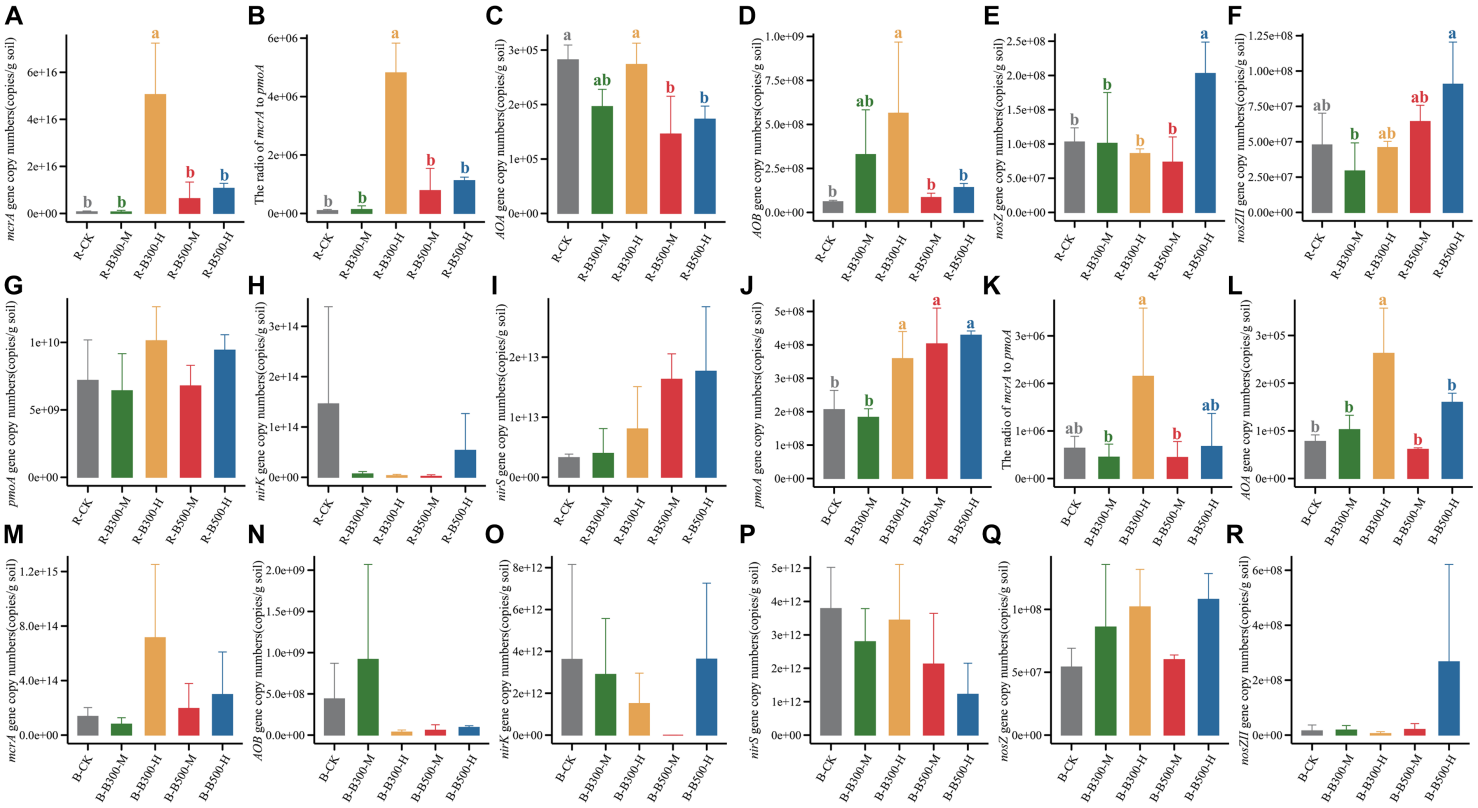
Abundance of CH_4_-related and N_2_O-related functional genes in rhizosphere and bulk soils. Panels **(A–I)** indicate the copy numbers of functional genes in rhizosphere soils. Panels **(J–R)** indicate the copy numbers of functional genes in bulk soils. Error bars indicate the standard deviation of triplicate analyses. Different lowercase letters indicate significant differences in different treatments (*p* < 0.05). No lowercase letters indicate no significant differences among different treatments (*p* > 0.05). The treatment labels are as follows: CK, without biochar addition; B300, biochar pyrolyzed at 300°C; B500, biochar pyrolyzed at 500°C; M, 2% (w:w) biochar addition; H, 10% (w:w) biochar addition; R, rhizosphere soil; B, bulk soil.

The abundance of methanogens and methanotrophs associated with CH_4_ emission was determined by qPCR. The *mcrA* gene copy number in the rhizosphere soil ranged from 1.26 × 10^13^ to 8.09 × 10^16^ (Figure 1), while in the bulk soil it ranged from 1.82 × 10^11^ to 1.38 × 10^15^ (Figure 1). The *pmoA* gene copy number in the rhizosphere soil ranged from 3.23 × 10^9^ to 1.37 × 10^10^ (Figure 1), while in the bulk soil it ranged from 1.34 × 10^8^ to 5.43 × 10^8^ (Figure 1). Compared with R-CK, the R-B300-H treatment significantly increased the *mcrA* gene copy number by 709.3% in rhizosphere soil. In the bulk soil, B-B300-H, B-B500-M, B-B500-H treatments significantly increased the *pmoA* gene copy number by 174%–208%. In both rhizosphere and bulk soils, the treatment of biochar at 300°C with 10% incorporation led to a significantly increased ratio of *mcrA*/*pmoA*.

The abundance of genes related to N_2_O emissions across various treatments in bulk and rhizosphere soils was assessed using qPCR (Figure 1). The abundance of the AOA gene ranged from 1.83 × 10^8^ to 4.29 × 10^8^ copies g^−1^ in rhizosphere soil and 6.13 × 10^4^ to 2.63 × 10^5^ copies g^−1^ in bulk soil (Figure 1). In rhizosphere soil, the addition of biochar at 500°C significantly decreased the abundance of the AOA gene compared with R-CK. In bulk soil, B-B300-H treatment significantly increased the abundance of the AOA gene. Compared with R-CK, R-B300-H treatment significantly increased the abundance of the AOB gene in rhizosphere soil (Figure 1). Comparably, the abundance of the AOB gene showed no difference in bulk soil with or without biochar amendment (Figure 1). In all treatments, no significant difference was found between the rhizosphere and bulk soils regarding *nirK* and *nirS* gene abundance (Figure 1). The abundance of *nosZ* and *nosZII* genes showed obvious differences only within the rhizosphere soil. No discernible differences were observed in the bulk soil across the various treatments (Figure 1). R-B500-H treatment significantly increased *nosZ* gene abundance compared with R-CK (Figure 1). Additionally, R-B300-M treatment significantly decreased *nosZII* gene abundance compared with R-CK, and a significant difference was observed between R-B300-M and R-B500-H (Figure 1). As a whole, the abundance of genes in rhizosphere soil was obviously higher than that in the bulk soil.

### 3.3. The structures of *pmoA* community

The *pmoA*-harboring microbial communities in the bulk and rhizosphere soils among different treatments were analyzed using Illumina MiSeq sequencing. The α diversity index was evaluated using Chao1, Coverage, Observed-species, Shannon, Simpson, and Pielou_e indices (Supplementary Table S3). The Chao1, Coverage, and Observed-species indices showed significant differences between rhizosphere and bulk soils. The Chao1 and Observed-species indices were higher in rhizosphere soil than in bulk soil, but the Coverage index was higher in bulk soil than in rhizosphere soil. In contrast, the Shannon, Simpson, and Pielou_e indices did not significantly change in any rhizosphere or bulk soil among the five treatments. NMDS of the soil *α* diversity index was indicated in Supplementary Figure S1A. The subsampling points for the rhizosphere and bulk soils treatments were notably distant from each other, indicating variations in the soil’s *α* diversity index among the different treatment groups. In the rhizosphere soil across different treatments, the subsample points of R-B500-M were far away from those of the R-B500-H treatment. This indicates that different amounts of biochar at 500°C significantly altered the *α* diversity index of rhizosphere soil. In the bulk soil, the distance between subsample points was consistently significant across different treatments, indicating that different types of biochar influenced the soil’s *α* diversity index.

**Figure 2:**
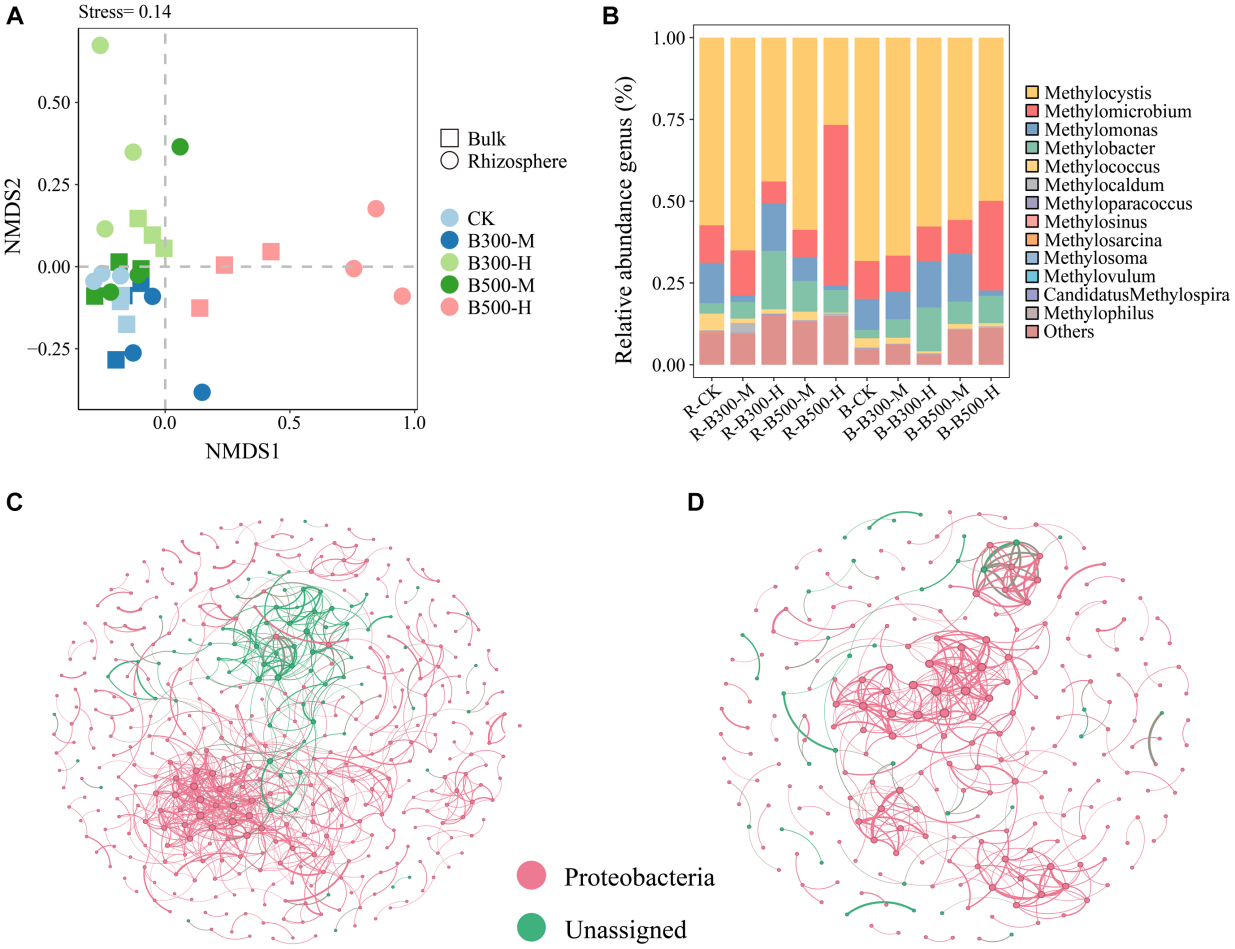
Non-metric multidimensional scaling analysis (NMDS) **(A)**, distribution of *pmoA* community composition **(B)**, and network analysis of *pmoA* community in rhizosphere **(C)** and bulk **(D)** soils. The treatment labels are as follows: CK, without biochar addition; B300, biochar pyrolyzed at 300°C; B500, biochar pyrolyzed at 500°C; M, 2% (w:w) biochar addition; H, 10% (w:w) biochar addition; R, rhizosphere soil; B, bulk soil.

NMDS of the methanotrophic community structure at the OTU level is indicated in Figure 2A. The NMDS results showed that methanotrophic community compositions in the rhizosphere soil significantly differed from those in the bulk soil of the control group, especially the treatments of biochar at 300°C and 10% incorporation, and biochar at 500°C and 10% incorporation (Figure 2A). In rhizosphere soil, the communities of methanotrophs exhibited dispersed distribution under four of the biochar treatments, indicating that the addition of biochar had a significant effect on their community structure. In bulk soil, the B-B300-M and B-B500-M treatments clustered together, indicating that these two treatments had a slight effect on their community structure (Figure 2A).

*Methylocystis* (72.16%–31.35%), *Methylomicrobium* (57.77%–8.05%), *Methylomonas* (17.04%–1.59%) and *Methylobacter* (21.14%–2.69%) were the most abundant methanotrophic communities in both rhizosphere and bulk soils (Figure 2B). The abundance of *Methylocystis* had a significant increase in rhizosphere and bulk soils in the treatment with 500°C biochar and 10% incorporation, whereas *Methylomicrobium* displayed the opposite trend (Figure 2B). In addition, with the 300°C biochar and 10% incorporation, the abundance of *Methylobacter* in rhizosphere and bulk soils increased significantly compared with the corresponding control treatments of R-CK and B-CK (Figure 2B).

Networks of the methanotrophic community were constructed to identify the cooccurrence relationship of microbial communities in rhizosphere and bulk soils. The number of nodes and edges (positive and negative correlations) were higher in the rhizosphere soil than in bulk soil. Among them, the number of positive correlations exceeded that of the negative correlations. Rhizosphere soil was more complex than bulk soil in their cooccurrence networks, indicating a higher degree of interaction among microbial communities in rhizosphere soil (Figures 2C,D).

### 3.4. The structures of *nosZ* community

The alpha-diversity of *nosZ*-containing microbes was analyzed (Supplementary Table S4) and the Observed-species index all decreased with biochar addition. As seen in the Chao1 index, only R-B300-H and B-B300-H significantly decreased the *α* diversity index, whereas, in Coverage index, R-B300-H and B-B300-H significantly increased the *α* diversity index compared to R-CK and B-CK. The *α* diversity index variation between samples was visualized using NMDS (Supplementary Figure S1B). The NMDS results showed that there was no obvious separation among the treatments between rhizosphere and bulk soils, indicating no significant differences between rhizosphere and bulk soils. In rhizosphere soil, subsample points of R-CK were notably distant from those of R-B300-H and R-B500-H treatments. This indicates that different temperatures of biochar at a 10% incorporation level significantly altered rhizosphere soil’s *α* diversity index. In the bulk soil, there was a substantial distance between B-CK and B-B300-H, indicating differences in the composition of the soil’s *α* diversity index.

**Figure 3:**
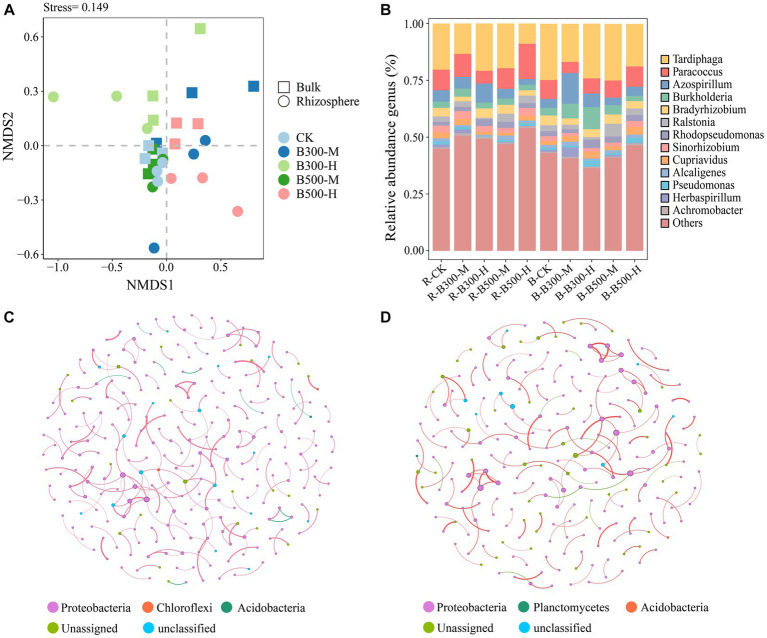
Non-metric multidimensional scaling analysis (NMDS) **(A)**, distribution of *nosZ* community composition **(B)**, and network analysis of *nosZ* community in rhizosphere **(C)** and bulk **(D)** soils. The treatment labels are as follows: CK, without biochar addition; B300, biochar pyrolyzed at 300°C; B500, biochar pyrolyzed at 500°C; M, 2% (w:w) biochar addition; H, 10% (w:w) biochar addition; R, rhizosphere soil; B, bulk soil.

NMDS was utilized to visualize the *β*-diversity of the *nosZ* community among different treatments (Figure 3A). The NMDS plots demonstrated that *nosZ* community compositions did not significantly change in the rhizosphere or bulk soil (Figure 3A). In rhizosphere soil, the subsample points of R-CK had the greatest distance from the R-B300-M, R-B300-H, and R-B500-H treatments, indicating significant disparities among those community structures. In bulk soil, B-CK, B-B300-M, and B-B500-M treatments showed a dispersed distribution pattern, indicating that biochar at a 10% incorporation level significantly affected the *nosZ* community structure (Figure 3A).

The most abundant *nosZ* genera in rhizosphere and bulk soils across the treatments were *Tardiphaga* (34.58%–55.16%), *Paracoccus* (11.26%–44.53%), *Azospirillum* (7.36%–31.25%), and *Burkholderia* (5.88%–20.21%; Figure 3B). In rhizosphere soil, the relative abundance of *Paracoccus* significantly decreased while that of *Azospirillum* significantly increased for the R-B300-H treatment compared to R-CK. Notably, the B-B300-M treatment showed similar changes in the bulk soil (Figure 3B).

Network analysis showed that the number of nodes and edges (positive and negative correlations) were approximately identical in the rhizosphere and bulk soils. Among them, the number of positive correlations was greater than negative correlations. As seen from the cooccurrence networks of rhizosphere and bulk soils, biochar addition has a slight effect on interactions among *nosZ* microbial communities (Figures 3C,D).

### 3.5. CH_4_ and N_2_O emissions

**Figure 4:**
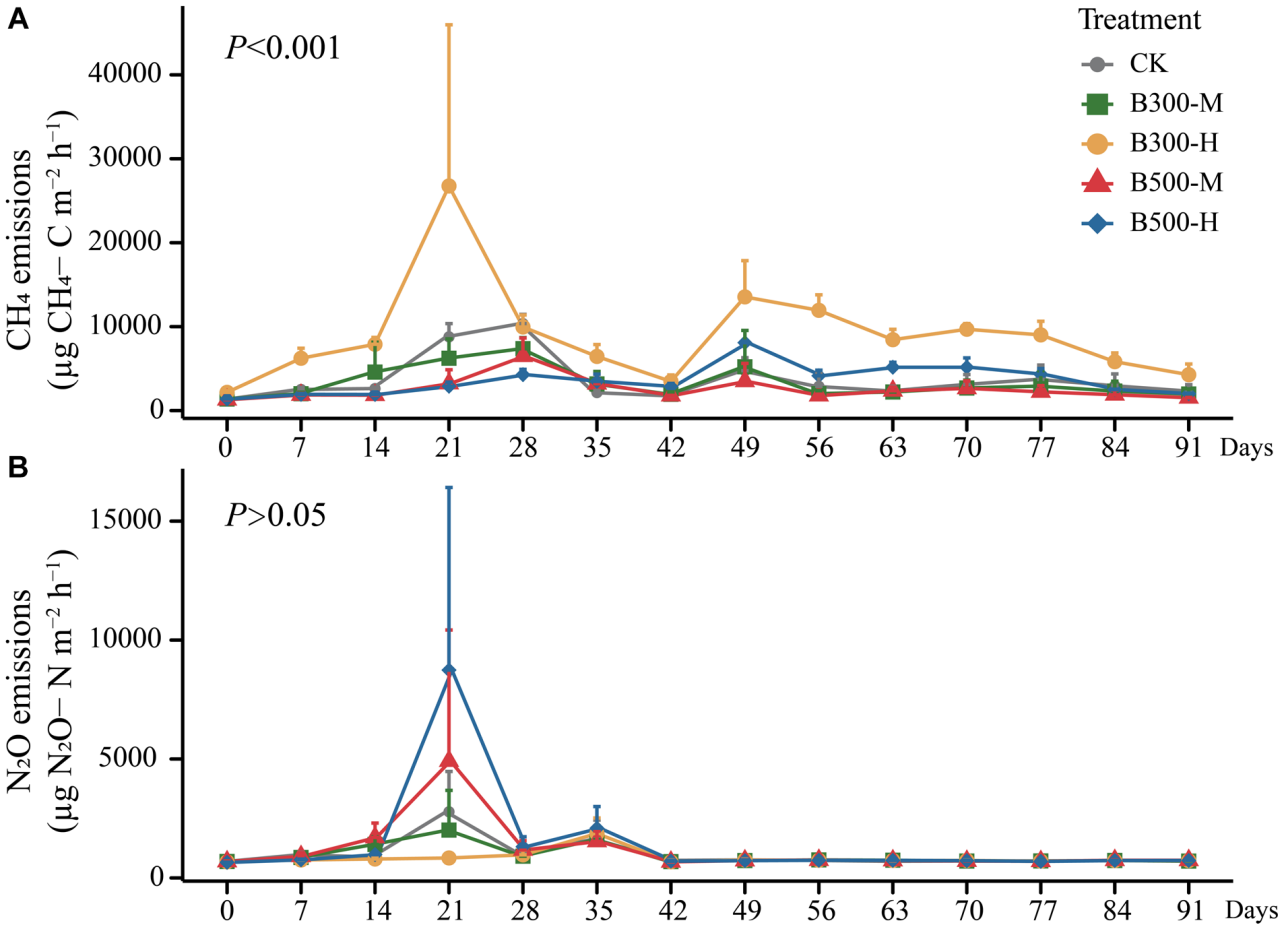
The cumulative emissions of CH_4_ **(A)** and N_2_O **(B)** from both rhizosphere and bulk soils during the growth of rice are displayed. Error bars indicate that the standard deviation of triplicate analyses. The treatment labels are as follows: CK, without biochar addition; B300, biochar pyrolyzed at 300°C; B500, biochar pyrolyzed at 500°C; M, 2% (w:w) biochar addition; H, 10% (w:w) biochar addition. The *p* value (*p* < 0.001) is for the significant effect on the CH_4_ emissions during the rice growth among different treatments. *p* > 0.05 indicates no significant effect on the N_2_O emissions during the rice growth among different treatments.

**Figure 5:**
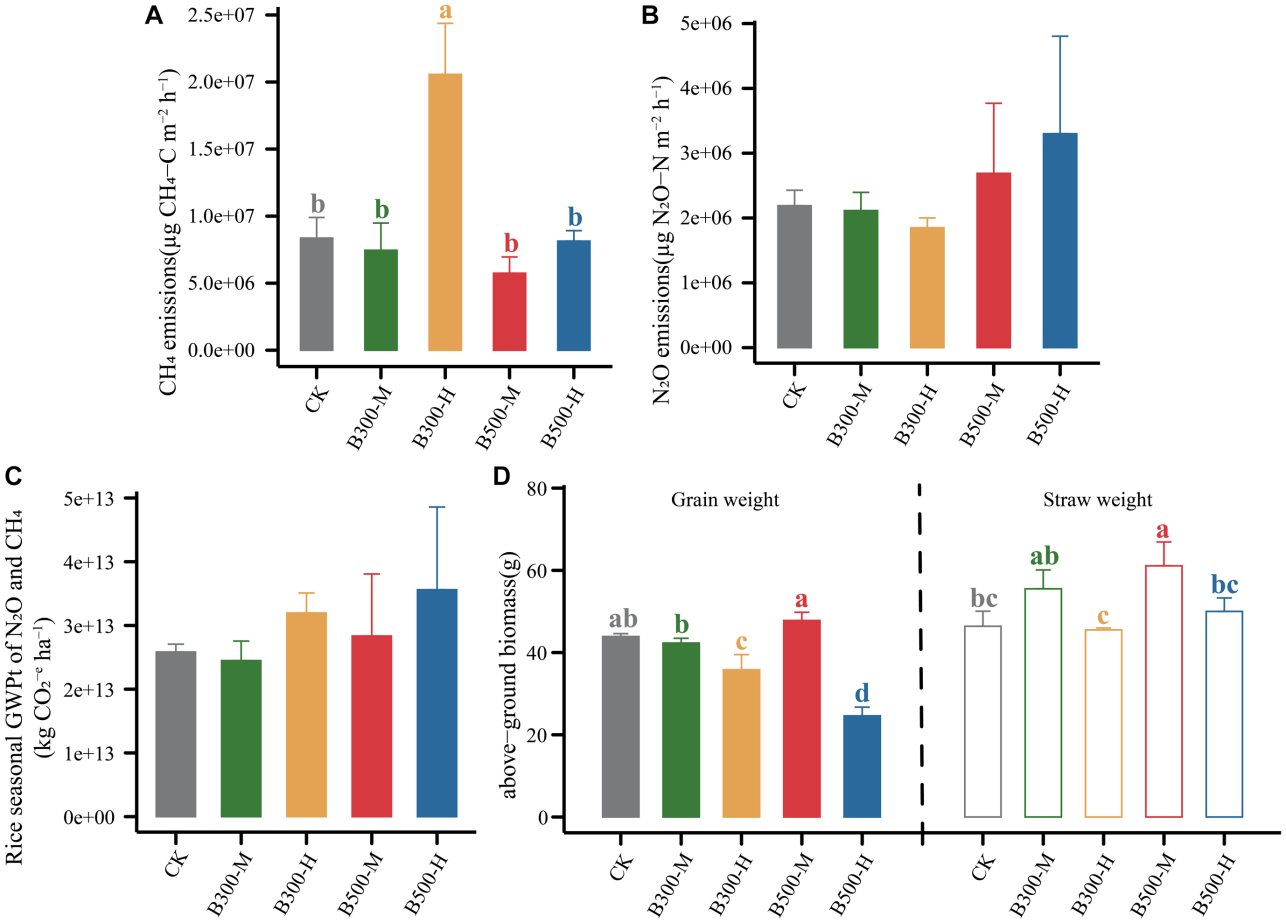
The cumulative emissions of CH_4_ **(A)** and N_2_O **(B)**, the total global warming potential (GWPt) **(C)** associated with N_2_O and CH_4_, and above-ground biomass **(D)** during the rice growth across different treatments are presented. Error bars indicate that the standard deviation of triplicate analyses. Different lowercase letters indicate significant differences in different treatments (*p* < 0.05). No lowercase letters indicate no significant differences among different treatments (*p* > 0.05). The treatment labels are as follows: CK, without biochar addition; B300, biochar pyrolyzed at 300°C; B500, biochar pyrolyzed at 500°C; M, 2% (w:w) biochar addition; H, 10% (w:w) biochar addition.

The CH_4_ emissions during the rice growing season are presented in Figure 4A. The results showed that highly significant differences were observed in CH_4_ fluxes among different treatments (*p* < 0.001). The B300-H treatment stimulated CH_4_ emissions during this period, with emission rates reaching their peaks at 21 days and 49 days (Figure 4A). There were no significant differences in the patterns of seasonal variation in CH_4_ fluxes among the remaining four treatments (Figure 4A). However, it is worth noting that biochar significantly increased cumulative CH_4_ emissions (only in the B300-H treatment) during the rice season (Figure 5A).

**Figure 6:**
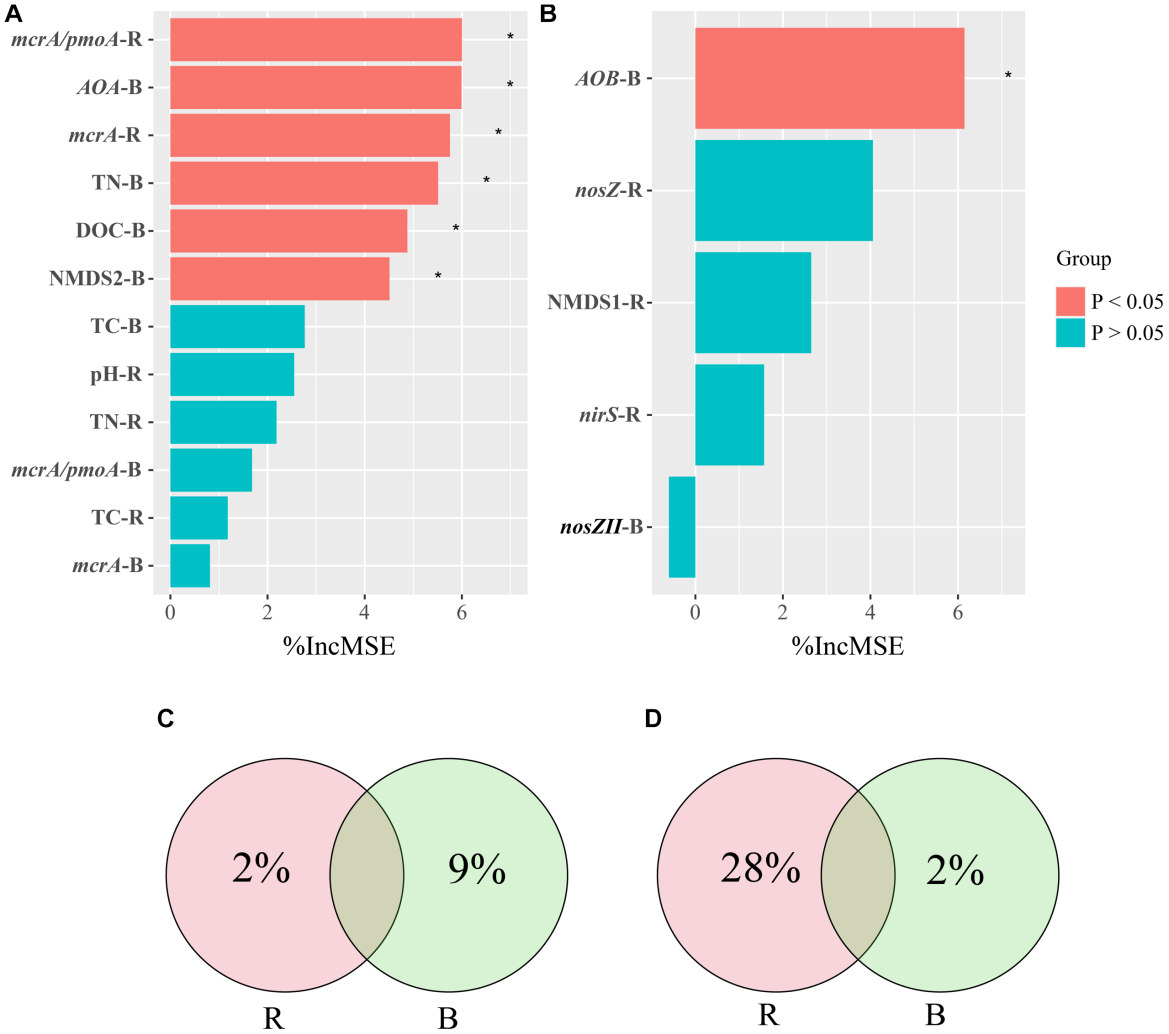
Random Forest Analysis against significant abiotic and biotic factors were analyzed for CH_4_ **(A)** and N_2_O **(B)** emissions. Variation Partitioning Analysis (VPA) against significant abiotic and biotic factors were analyzed for CH_4_ **(C)** and N_2_O **(D)** emissions. R, rhizosphere soil; B, bulk soil.

Correlations among soil properties, the abundance of genes, the microbial community of *pmoA,* and CH_4_ emissions were calculated using Pearson correlation analysis (Supplementary Figure S2). We screened for factors associated with CH_4_ emissions in rhizosphere and bulk soils and performed a random forest analysis. Random forest regression analyses revealed that several important factors influenced CH_4_ emissions. In rhizosphere soil, *mcrA/pmoA* and *mcrA* played significant roles, while in bulk soil, *AOA*, TN, DOC, and NMDS2 were notable contributors (Figure 6). Specifically, the variables *mcrA/pmoA* in rhizosphere soil and *AOA* in bulk soil were particularly significant, contributing 6% of the overall importance in affecting CH_4_ emissions. We quantified the holistic effect of rhizosphere and bulk factors on CH_4_ emissions through variance partitioning analysis (VPA). The VPA results showed that the pure effects of rhizosphere and bulk factors were around 2, and 9%, respectively (Figure 6), in which bulk factors contributed more than rhizosphere factors.

All treatments showed similar variational patterns in the N_2_O fluxes throughout the entire rice growing season (Figure 4B). For the N_2_O fluxes, the highest peak was at 21 days. After 21 days, the N_2_O fluxes showed significant differences in all treatments. Compared with CK, biochar addition at 500°C significantly increased N_2_O fluxes, while biochar addition at 300°C reduced N_2_O fluxes. In all treatments, the N_2_O fluxes remained at a low rate until the rice was harvested (Figure 4B). None of the biochar treatments had a remarkable impact on N_2_O emissions (Figure 5B). Correlation analysis showed that the abundance of *AOB* and *nosZII* in bulk soil and the abundance of *nirS* and *nosZ* in rhizosphere soil was strongly correlated with N_2_O emissions (Supplementary Figure S3). The NMDS1 of *nosZ* in rhizosphere soil positively correlated with N_2_O emissions (Supplementary Figure S3). Random forest regression analyses revealed that at least 6% importance of *AOB* in bulk was significant for impacting N_2_O emissions (Figure 6). We quantified the holistic effect of rhizosphere and bulk factors on N_2_O emissions through VPA analysis. VPA results showed pure effects of rhizosphere and bulk factors were around 28, 2%, respectively (Figure 6), which rhizosphere factors contributed more than bulk factors.

### 3.6. GWPt and crop yield

The GWPt was calculated based on CH_4_ and N_2_O emissions. There was no significant difference in the GWPt in the five treatments (Figure 5C). Biochar pyrolyzed at 300°C and applied at a 2% level (B300-M) mitigated the GWPt, and the remaining three treatments (B300-H, B500-M, B500-H) increased the GWPt (Figure 5C). Figure 5D shows the variation in rice yield among different treatments. Biochar at a 10% incorporation level in the treatments of B300-H and B500-H significantly decreased the grain yield. However, applying biochar at a 2% incorporation level in the treatments of B300-M and B500-M had no significant effect on the grain yield. Furthermore, the B500-M treatment significantly increased the straw weight compared with CK (Figure 5D).

## 4. Discussion

### 4.1. Effect of biochar incorporation on soil physicochemical properties

Our studies showed that biochar could significantly change soil physicochemical properties such as pH, NH_4_^+^-N and DOC, which was consistent with previous studies (Alkharabsheh et al., 2021; Li et al., 2021; Table 1). Additionally, we also found that there were noteworthy distinctions in physiochemical properties between rhizosphere and bulk soils (Table 1). These differences of physiochemical properties between rhizosphere and bulk soils could be attributed to the influence of rice plant growth, which changed rhizospheric soil properties through root exudation and the release of oxygen (Jones et al., 2009; Breidenbach et al., 2015). We found that the pH values in both rhizosphere and bulk soils were significantly higher in biochar treatments than in control treatment. This may be due to the fact that biochar itself is strongly alkaline (Kubaczyński et al., 2022). And the pH in rhizosphere soil was higher than that in bulk soil. This indicated that the rhizosphere soil was more responsive to biochar. Compared to control, 2% biochar application decreased the content of NH_4_^+^-N, while 10% biochar application increased the content of NH_4_^+^-N in both rhizosphere and bulk soils. Vahidi et al. (2022) found that high levels of biochar application was more beneficial in increasing soil nutrients, which was consistent with our findings. DOC is an important indicator of soil nutrients. We found that biochar pyrolyzed at 300°C treatments increased DOC content, while biochar pyrolyzed at 500°C treatments decreased it in both rhizosphere and bulk soils. This is probably because biochar pyrolyzed at 300°C contained a higher concentration of DOC compared to biochar pyrolyzed at 500°C (Nan et al., 2022). Similarly, we found rhizosphere soil had higher levels of NH_4_^+^-N and DOC than bulk soil. This indicated that biochar application had a greater impact on nutrient accumulation in the rhizosphere soil than that in bulk soil.

### 4.2. Effect of biochar incorporation on soil microbial diversity and community structures

Soil *α* diversity in both *pmoA* and *nosZ* communities exhibited significant alterations across different treatments (Supplementary Tables S3, S4). For both *pmoA* and *nosZ* communities, it was observed that the *α* diversity in rhizosphere soil exceeded that in bulk soil, largely attributable to the rhizosphere effect. It is worth emphasizing that soil properties were important factors in shaping microbial community composition (Zhalnina et al., 2015; Ji et al., 2021). The rhizosphere effect, driven by rice plant growth, has the capacity to induce changes in soil properties, thereby altering the microbial community. However, the *nosZ* communities’ Chao1 index of biochar addition at a 10% incorporation level led to a significant reduction in *α* diversity in rhizosphere and bulk soils, when compared to the corresponding control treatments of R-CK and B-CK. This finding suggests that excessive use of biochar might have detrimental impact on the diversity of soil bacterial communities (Wang et al., 2020).

The NMDS results revealed significant differences in the *pmoA* community structure between rhizosphere and bulk soils (Figure 2). Furthermore, a higher rate of biochar application had a more pronounced influence on shaping the bacterial communities within rhizosphere soil (Figure 2A). The variations of *pmoA* community composition in both rhizosphere and bulk soils were more obvious in the treatment with 10% biochar incorporation when compared to the 2% biochar incorporation and the corresponding control treatments of R-CK and B-CK (Figures 2A,B). The rhizosphere effect can alter the soil properties (Sun P. et al., 2021), thus there were significant differences between rhizosphere and bulk soils. The greater effect of adding 10% biochar on composition of *pmoA* community could be attributed to the fact that substantial alterations in soil microbial communities only occurred when a certain threshold of biochar was present in the soil. This aligns with the conclusions of Amoakwah et al. (2022). Similar to the *pmoA* community, biochar at a 10% incorporation significantly altered the structure of the *nosZ* community in both rhizosphere and bulk soils compared to the corresponding control treatments of R-CK and B-CK (Figures 3A,B). This effect may be ascribed to the enhancement of the soil environment due to the biochar addition, which impacts the composition of the soil microbial community (Lehmann, 2007; Farrell et al., 2013). This effect may also be ascribed to the porous nature of biochar, which provides a suitable microenvironment for the microbial community to survive (Shi et al., 2022).

Network analysis helps to identify the microbial taxa that have the greatest impact on the microbial community and reveal the correlations among microorganisms (Guseva et al., 2022). In regard to *pmoA* communities, significant differences were observed between rhizosphere and bulk soils. The number of nodes and edges was higher in rhizosphere soil in comparison to bulk soil (Figures 2C,D), indicating that links within the *pmoA* communities in bulk soil were considerably fewer when compared to the present within rhizosphere soil. This may be attributed to the higher number of microorganisms in rhizosphere soil than in bulk soil (Yuan et al., 2023). The presence of more positive and negative links in rhizosphere soil compared to bulk soil suggests that rhizosphere soil have positive effects on the interactions between synergistic and antagonistic taxa (Zhu et al., 2022). In *nosZ* communities, the complexity of the soil microbial community network was higher in rhizosphere soil than in the bulk soil (Figures 3C,D), which indicated that the *nosZ* communities in the rhizosphere soil had better ability to maintain the stability of the microbial network than that in the bulk soil (Chen L. et al., 2019).

### 4.3. Effect of biochar incorporation on CH_4_ emissions

B300-H treatment significantly increased CH_4_ emissions (Figure 5A). This increase could be attributed to the higher abundance of *mcrA* and the ratio of *mcrA/pmoA* in both rhizosphere and bulk soils of the B300-H treatment (Figure 1). In a long-term field observation of biochar amendment conducted by Wu et al. (2023), it was found that CH_4_ emissions were increased with biochar addition in the first year. This initial increase can be attributed to the fact that biochar application provided substrates for methanogens in the short term. Compared to biochar pyrolyzed at 500°C, biochar pyrolyzed at 300°C contained a higher concentration of DOC and served as carbon substrates to enhance methanogenic activity (Nan et al., 2022), which aligns with the observation that DOC levels were higher in the B300-H treatment compared to the other treatments in bulk soil (Table 1). Correlation analysis between soil properties and the abundance of functional genes showed a highly positive correlation between pH and the abundance of *mcrA* (Supplementary Figure S4). The results indicated that pH significantly affected the abundance of *mcrA* in rhizosphere soil. This is consistent with the study by Le Mer and Roger (2001), which showed that the increase in soil pH triggered the growth of methanogens.

In contrast, the B500-H treatment did not lead to a significant increase in CH_4_ emissions when compared with B300-H. This disparity may be attributed to the unique properties of high-temperature biochar, characterized by its fused aromatic ring structure and redox activity due to the presence of quinone and hydroquinone structure, thereby these properties mitigate CH_4_ emissions (Sun T. et al., 2021; Dong et al., 2023). Some studies showed that biochar favored electron transfer between bacteria and iron minerals, dissimilatory Fe(III) reducing bacteria, which commonly compete with methanogens for electron donors in anoxic environments, can thus contribute to reducing CH_4_ emissions (Wang et al., 2017; Sun T. et al., 2021; Dong et al., 2023). Correlation analysis showed that a significant correlation existed between the abundance of *AOA* in bulk soils and CH_4_ emissions (Supplementary Figure S2). Moreover, the random forest analysis showed the ratio of *mcrA/pmoA* in rhizosphere soil and the abundance of *AOA* in bulk soil were the most important factors impacting CH_4_ emissions (Figure 6). Some studies have shown that the role of *AOA* is important in CH_4_ oxidation due to their high ammonia/CH_4_ affinity (Stein et al., 2012; Bodelier and Steenbergh, 2014). Furthermore, the findings of Xu et al. (2021) showed that *AOA* could influence CH_4_ emissions through their interactions with methanogens and methanotrophs.

In order to explore the extent to which rhizosphere and bulk soils influence CH_4_ emissions, we used VPA (Figure 6C). The VPA results show that among the many factors associated with CH_4_ emissions, the bulk soil factors contribute more to CH_4_ emissions than the rhizosphere soil factors. We conclude that bulk soil plays a greater role in CH_4_ production. Some studies showed that biochar application has the potential to mitigate CH_4_ production by increasing activities of methanotrophs in the rhizosphere (Nguyen et al., 2020; Lehmann et al., 2021). This is consistent with the findings of Wu et al. (2019), which found that CH_4_ produced by paddy soil can be oxidized in the relatively oxygen-rich in rhizosphere due to the presence of aerobic methanotrophs. However, this particular study found that factors in bulk soil had a greater impact on CH_4_ emissions compared to rhizosphere soil. We speculate that the flooding of rice fields during growth likely creates anaerobic conditions where bulk soil factors play a dominant role in CH_4_ emissions under anaerobic conditions.

### 4.4. Effect of biochar incorporation on N_2_O emissions

Correlation analysis shows that there was a significant correlation between *nosZ* and *nirS* in rhizosphere soil, *AOB* and *nosZII* in bulk soil, and N_2_O emissions (Supplementary Figure S3). *NirS* and *nosZ* are core functional genes for denitrification processes in the N cycle (Zhao et al., 2019). The abundance of *nirS* and *nosZ* genes were higher in rhizosphere soil than in bulk soil. The results showed great differences in denitrification activities between rhizosphere and bulk soils, which is consistent with Scheer et al. (2008). Additionally, Baudoin et al. (2003) found that root exudates could be used as C sources for the growth of denitrifying bacteria, thereby increasing denitrification activity. Moreover, studies have identified the predominant role of *nirS*-type denitrifiers in rhizosphere soil, underscoring their significant influence on N_2_O production (Ai et al., 2017). Besides abundance levels, there was a significant correlation between NMDS1, which stands for the rhizosphere denitrifiers based on the *nosZ* gene, and N_2_O emissions. This finding suggested that biochar addition could influence N_2_O emissions by altering the structure of *nosZ* communities. Among them, the abundance of *Paracoccus* in rhizosphere showed a positive correlation with N_2_O emissions (Supplementary Figure S5). Notably, the *Paracoccus*, a member of the Proteobacteria, is recognized for its significant role in N_2_O emissions (Zhang et al., 2022).

In bulk soil, there was a significant correlation between the abundance of *AOB* and *nosZII* and N_2_O emissions. In order to determine the degree of contribution to N_2_O emissions among different factors, we performed a random forest analysis. The results showed that the abundance of *AOB* in bulk soil was the most important factor affecting N_2_O emissions (Figure 6B). We found that the abundance of genes (*nosZ*, *nirS*) in rhizosphere soil had a significant impact on N_2_O emissions in this study (Figure 6D). The abundance of genes in rhizosphere soil could explain more than 30% of the variations in N_2_O emissions (Figure 6D). The VPA results showed that rhizosphere soil has a greater influence on N_2_O emissions than bulk soil, which is consistent with the findings from Xing et al. (2021) that microelectrodes were employed to measure N_2_O emissions from agricultural rhizosphere and bulk soils, revealing significantly higher N_2_O emissions from the rhizosphere soil in comparison to the bulk soil.

### 4.5. Effects of biochar incorporation on rice growth

In this study, biochar amendment significantly improved rice growth in the B500-M treatment (Figure 5D), which was consistent with the previous study that biochar application could increase crop yields (Agegnehu et al., 2016). Correlations analysis among soil properties, the abundance of functional genes, and crop yield indicated that there was a significantly negative correlation between grain weight and soil N content (NH_4_^+^-N, NO_3_^−^-N, TN), TC, *nosZ* abundance in rhizosphere soil (Supplementary Figure S4). In bulk soil, grain weight was significantly and negatively correlated with soil N content (NH_4_^+^-N, TN), the abundance of *nosZ*, *nirK* and *AOA* (Supplementary Figure S6). This is consistent with the findings of Khan et al. (2022) that biochar could influence crop yield by improving soil properties and altering functional microbial abundance.

In future studies, gas emissions can be accurately assessed separately in rhizosphere and bulk soils. We found that applying biochar at 300°C and 10% incorporation not only increased the greenhouse gas emissions, but also reduced crop yield (Figure 5). In contrast, the treatment of biochar at 500°C and 2% incorporation not only increased grain yield but also did not lead to an increase in greenhouse gas emissions. Taking all factors into consideration, the treatment of biochar at 500°C and 2% incorporation can be considered a suitable choice for actual farmland production.

## 5. Conclusion

Biochar application significantly altered the soil properties in rhizosphere and bulk soils. The abundance of functional genes was higher in rhizosphere soil than in bulk soil with or without biochar incorporation. The composition of the soil microbial community also exhibited significant differences among the five treatments in rhizosphere and bulk soils. In particular, rhizosphere soil showed closer interactions among microbial communities than bulk soil at *pmoA*. There were significant differences in *α* diversity index between rhizosphere and bulk soils. This indicates that biochar application significantly altered soil properties, enhanced the abundance of functional genes, and altered the structure of *pmoA* and *nosZ* communities in the bulk and rhizosphere soils. In addition, the increased ratio of *mcrA/pmoA* in rhizosphere soil and the abundance of *AOA* in bulk soil may be the essential factors that lead to the increased CH_4_ production in soils. The VPA shows that the abundance of genes (*nosZ*, *nirS*) in the rhizosphere soil has a greater influence on N_2_O emissions than that in bulk soil. Bulk soil has a greater impact on CH_4_ production and rhizosphere soil has a greater impact on N_2_O production. Compared to the treatment of biochar at 300°C and 10% incorporation, biochar at 500°C and 2% incorporation mitigated greenhouse gas emissions and guaranteed a higher rice yield. In conclusion, we recommend the application of biochar at 500°C and 2% incorporation for farm production.

## Data availability statement

The datasets presented in this study can be found in online repositories. The names of the repository/repositories and accession number(s) can be found in the article/Supplementary material.

## Author contributions

J-QQ: Conceptualization, Data curation, Formal analysis, Investigation, Methodology, Writing – original draft. H-YY: Conceptualization, Data curation, Formal analysis, Funding acquisition, Investigation, Methodology, Supervision, Writing – review & editing. Q-LZ: Conceptualization, Investigation, Methodology, Writing – review & editing. E-FZ: Writing – review & editing. X-FT: Writing – review & editing. B-XT: Funding acquisition, Writing – review & editing. B-HZ: Writing – review & editing.
