# Supplementary material for: Effect of different types of biochar on soil properties and functional microbial communities in rhizosphere and bulk soils and their relationship with CH4 and N2O emissions
Source: Front Microbiol. 2023 Nov 2;14:1292959. doi: 10.3389/fmicb.2023.1292959 (PMC10656817; doi:10.3389/fmicb.2023.1292959)
Supplement: Supplementary file 1 [file Data_Sheet_1.docx]

Supplementary Material


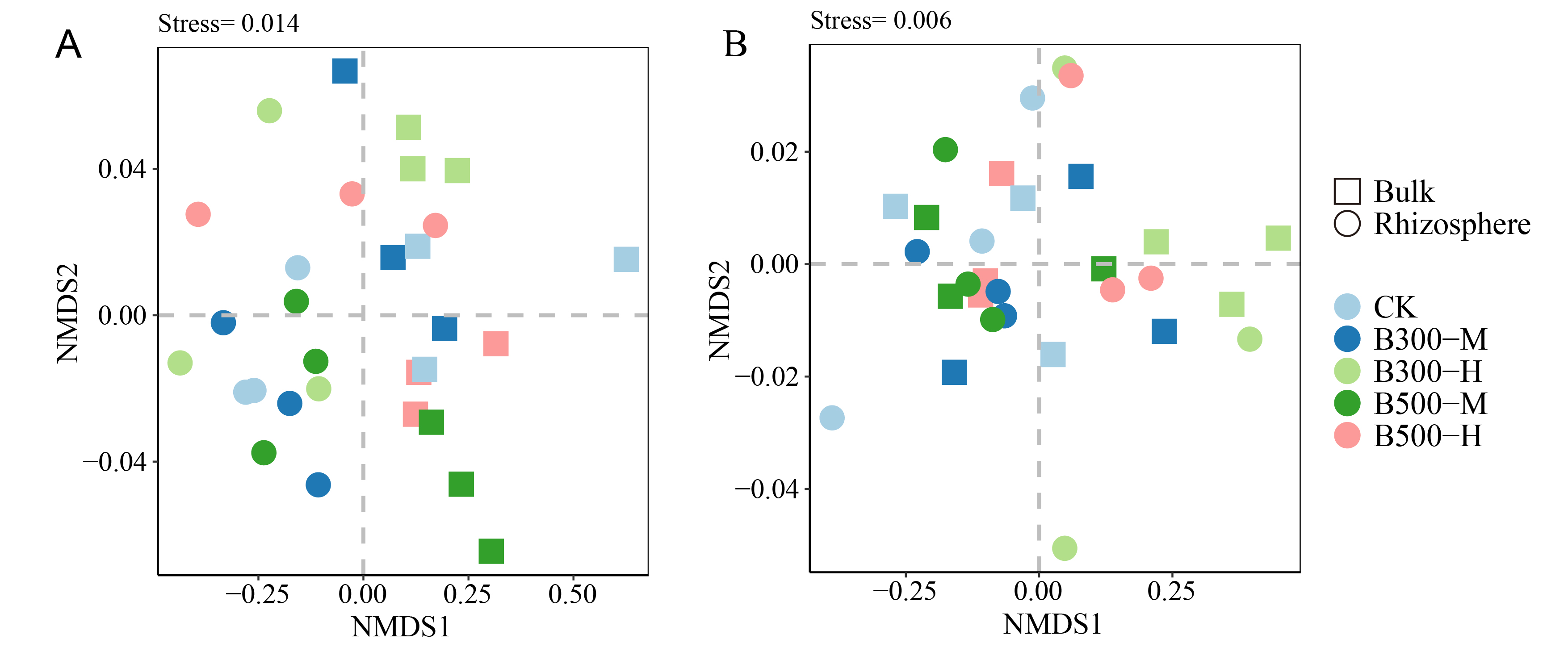


**Fig. S1** Non-metric multidimensional scaling analysis (NMDS) of α diversity of *pmoA* (A) and *nosZ* (B) communities among the different treatments. The treatment labels are as follows: CK, without biochar addition; B300, biochar pyrolyzed at 300°C; B500, biochar pyrolyzed at 500°C; M, 2% (w:w) biochar addition; H, 10% (w:w) biochar addition.


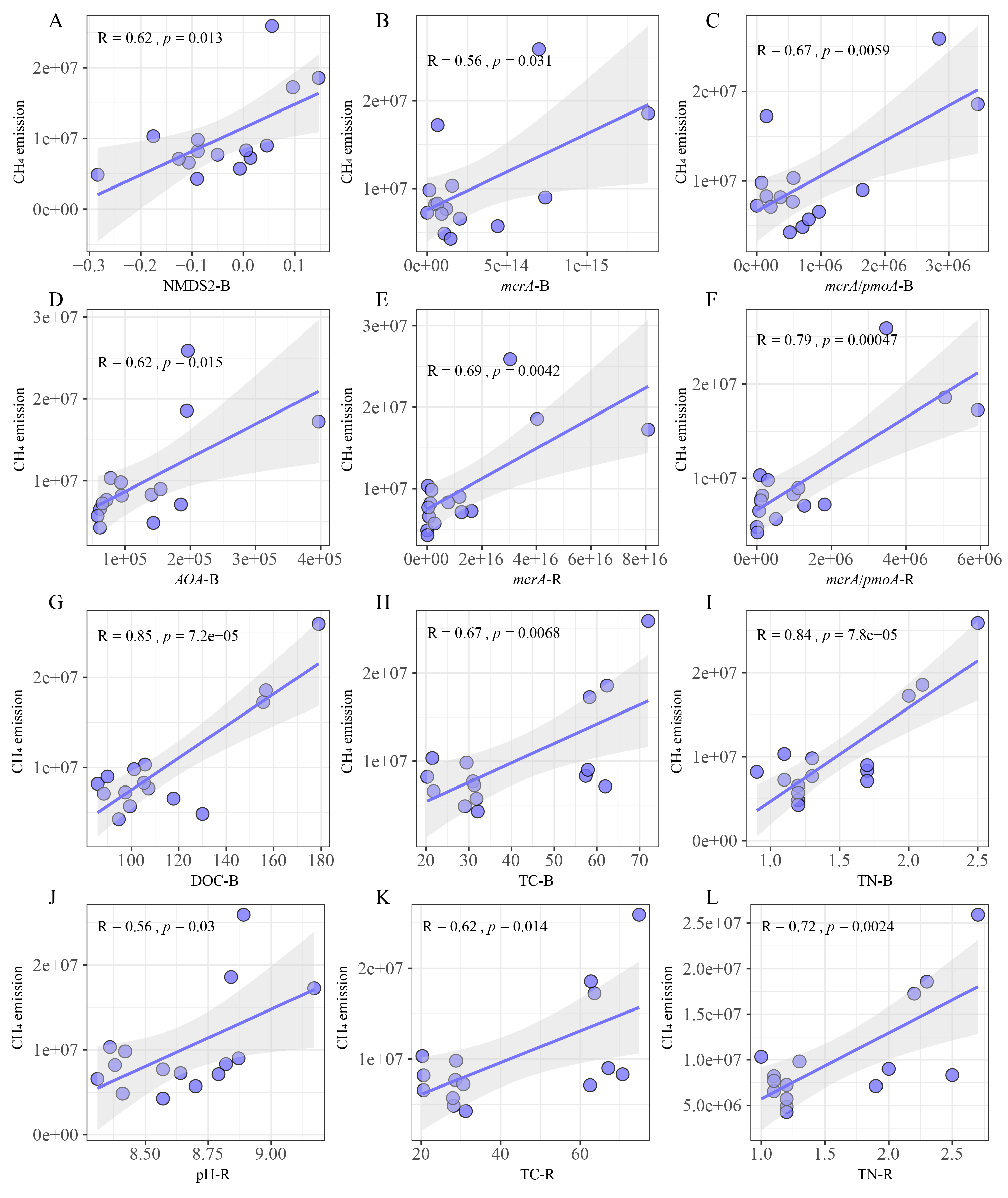


**Fig. S2** The relationships between the different factors in rhizosphere and bulk soils with CH_4_ emissions (*P*<0.05). R, rhizosphere soil; B, bulk soil.


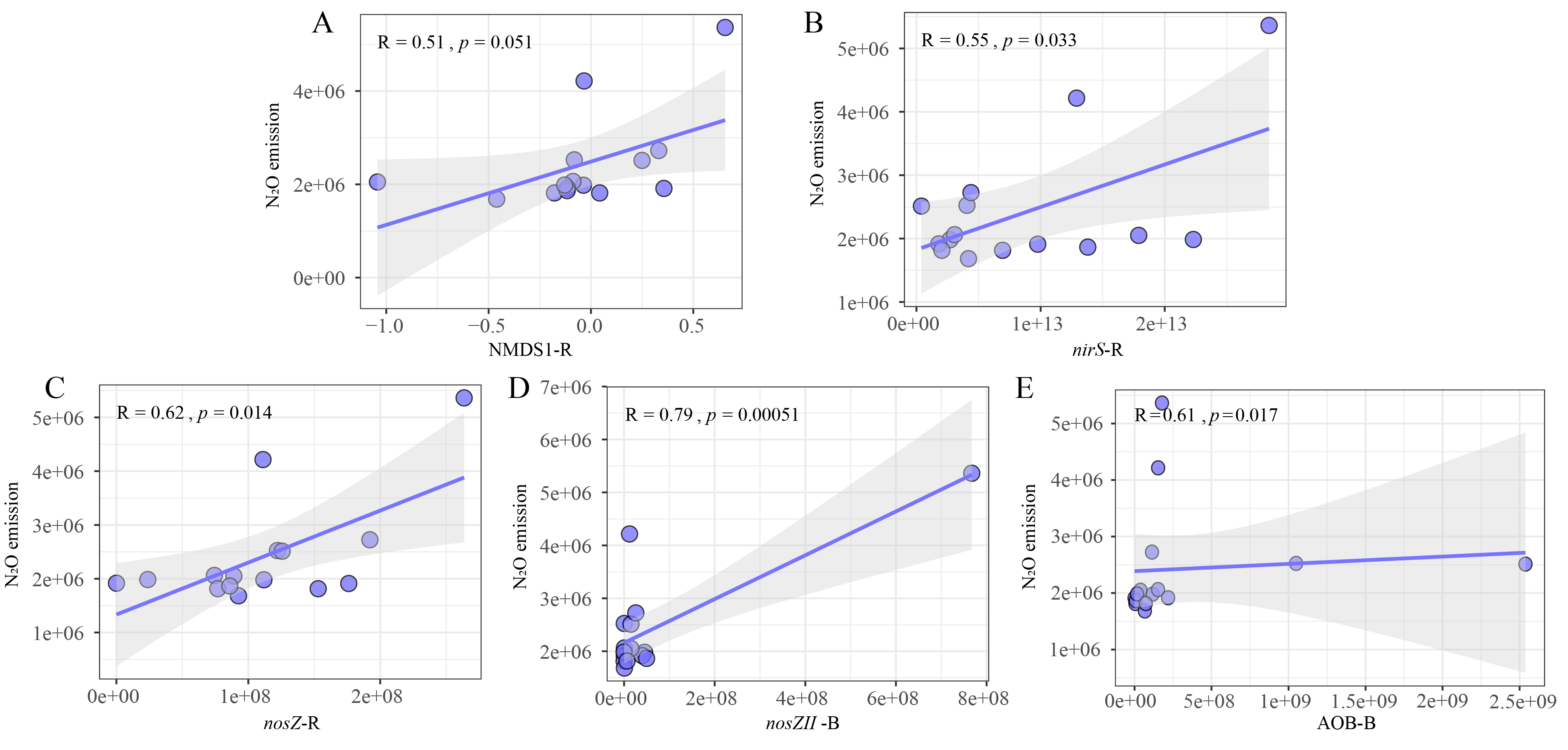


**Fig. S3** The relationships between the different factors in rhizosphere and bulk soils with N_2_O emissions (*P*<0.05). R, rhizosphere soil; B, bulk soil.


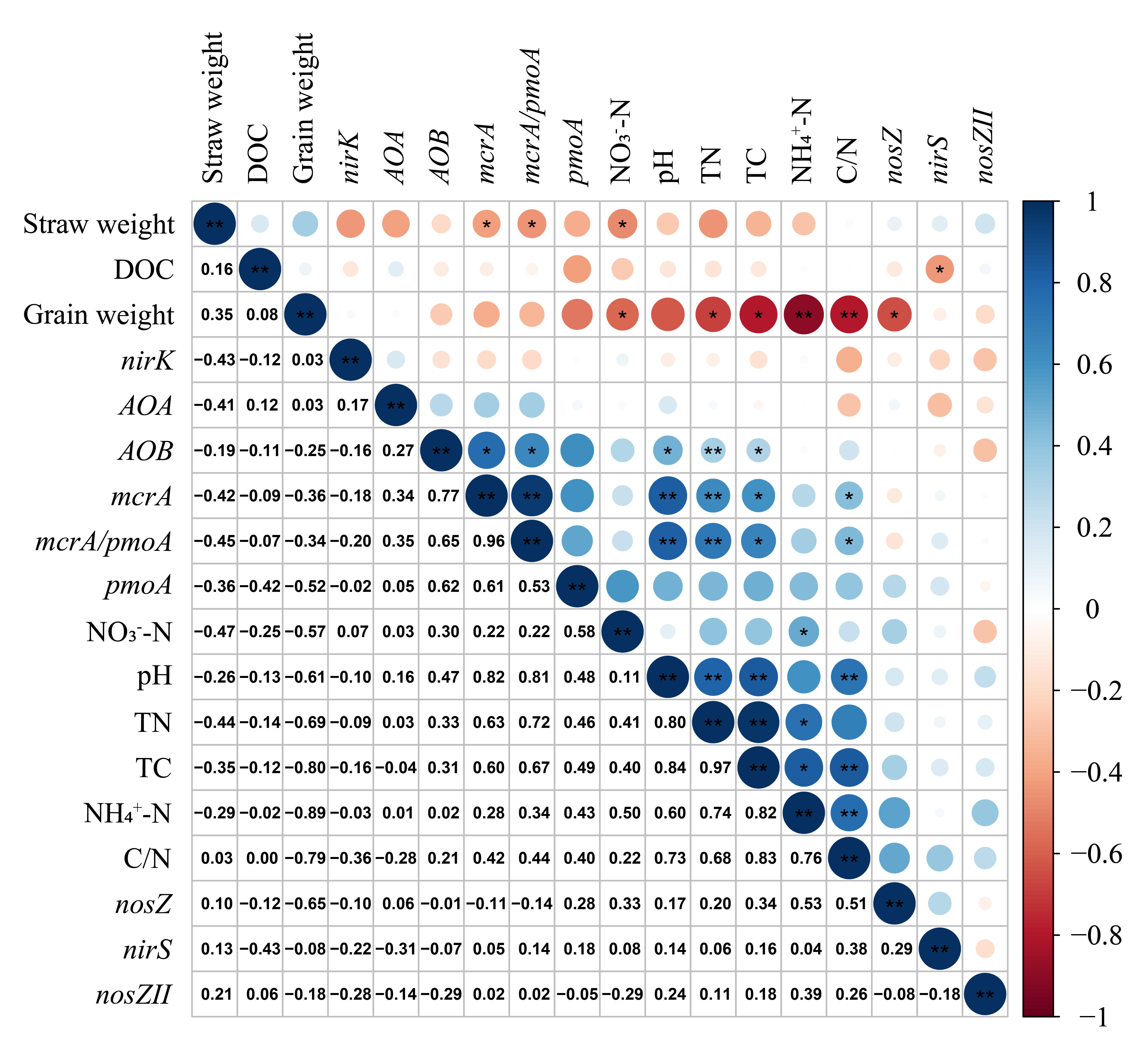


**Fig. S4** Correlations among soil physicochemical properties, the abundance of functional genes, and straw and grain weight in rhizosphere soil (*P* < 0.05).


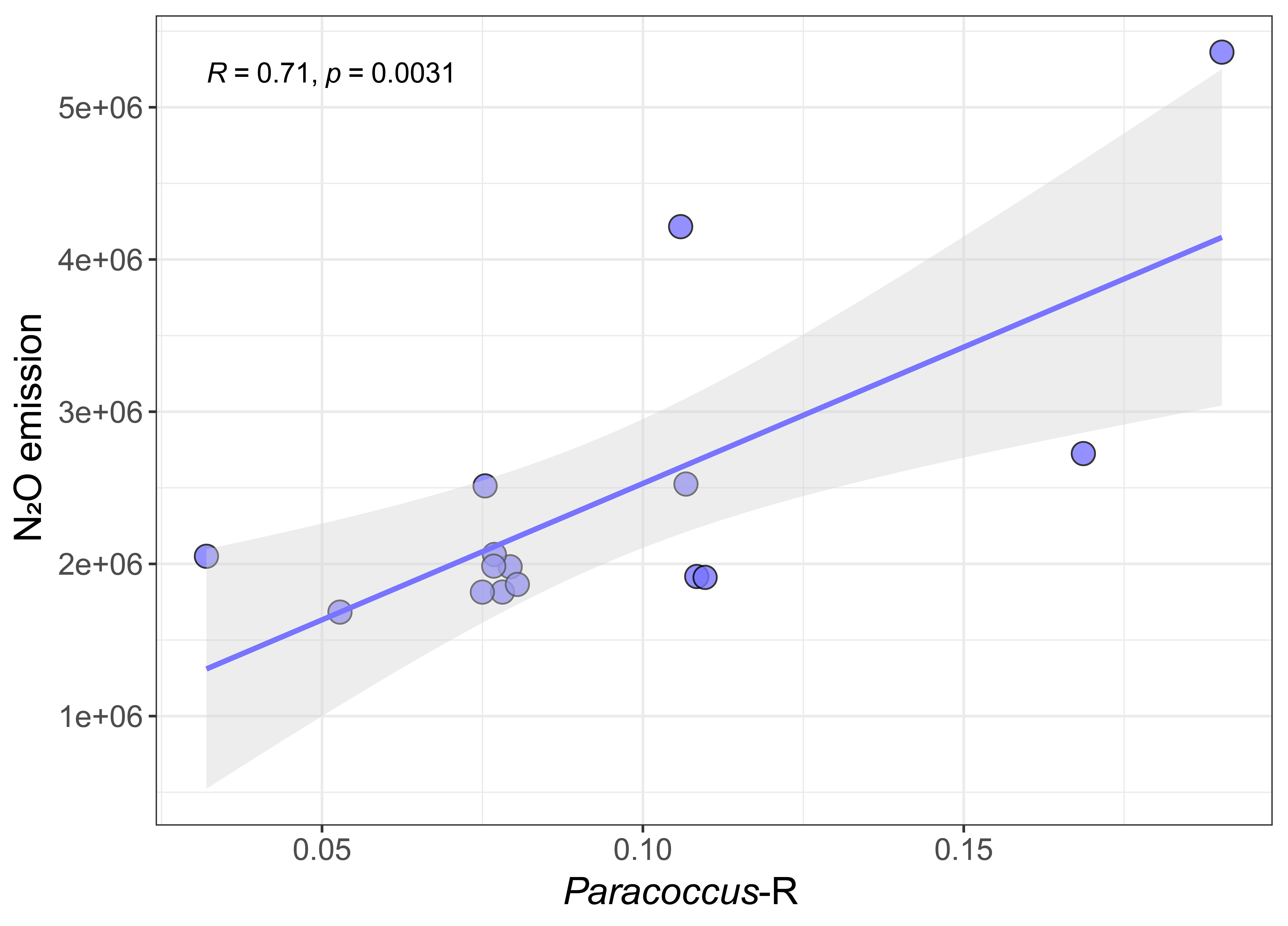


**Fig. S5** The relationship between the abundance of *Paracoccus* in rhizosphere soil and N_2_O emissions (*P*<0.05).


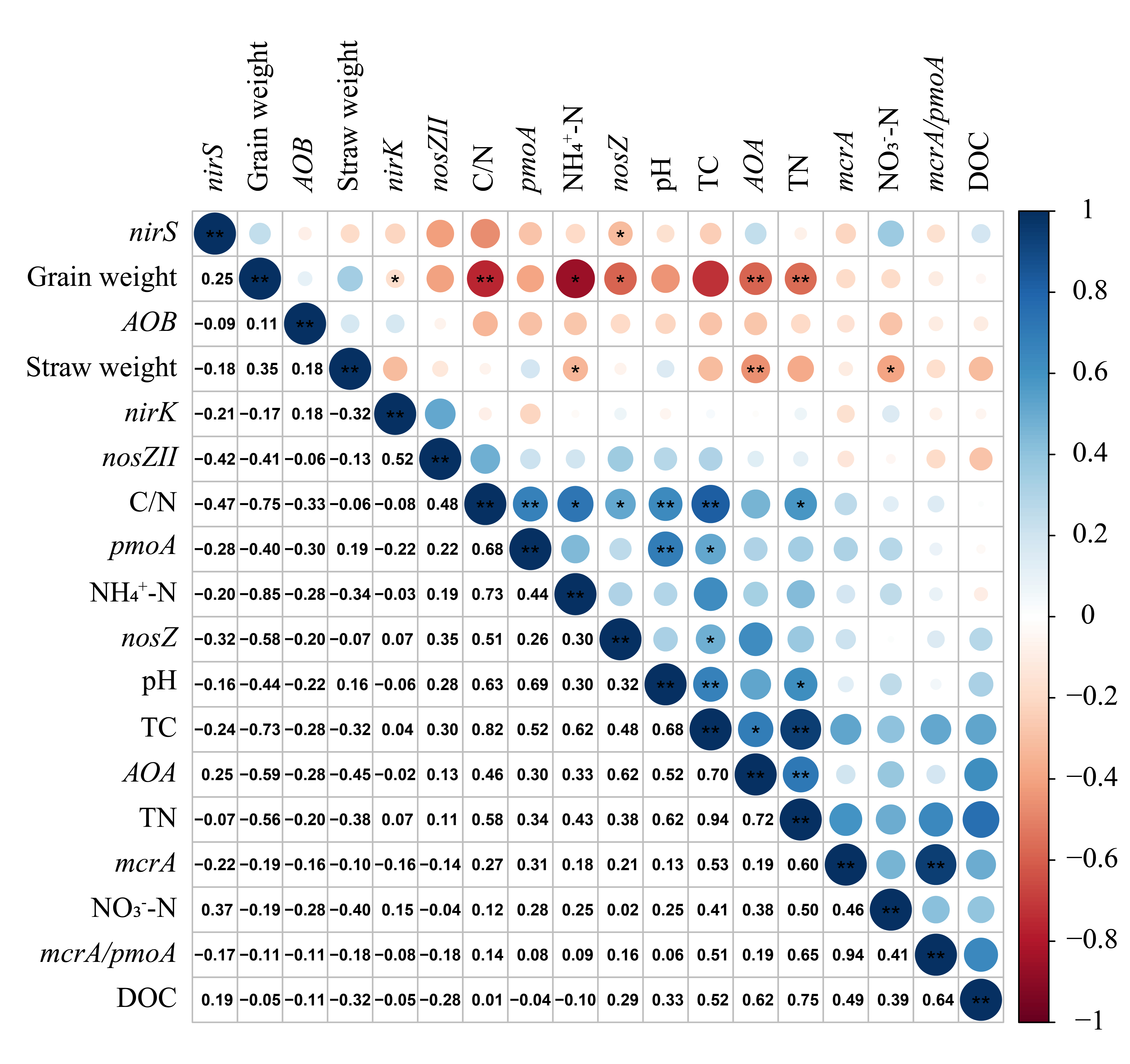


**Fig. S6** Correlations among soil physicochemical properties, the abundance of functional genes, and straw and grain weight in bulk soil (*P* < 0.05).

**Table S1** Original soil physiochemical properties

| Treatment | pH | NO_3_^-^-N(mg/kg) | NH_4_^+^-N(mg/kg) | DOC(mg/kg) |
| --- | --- | --- | --- | --- |
| soil | 8.22±0.03 | 721.79±9.56 | 16.66±6.69 | 498.84±17.83 |

Data are represented by their means and standard deviation (n = 3). Lowercase letters indicate significant differences among different treatments (*P*<0.05).

**Table S2** Details of primer sets and thermal conditions used in the qPCR assays

| Target genes | Primers | Sequence (5'-3') | Length of fragments | Thermal profile | References |
| --- | --- | --- | --- | --- | --- |
| *mcrA* | ME1  ME2 | GCMATGCARATHGGWATGTC  TCATKGCRTAGTTDGGRTAGT | 719 | 94°C for 3min; 94℃ for 45s, 50℃ for 45s, 72℃ for 90s and plate read at 83 ℃ for 10s (35cyles) | (BA et al., 1996) |
| *pmoA* | A189f  mb661r | GGNGACTGGGACTTCTGG  CCGGMGCAACGTCYTTACC | 491 | 94°C for 2min; 94℃ for 45s, 53℃ (a touchdown from 58℃ to 52℃, -1.0℃,6 circles) for 1min, 50℃ for 45s, 72℃ for 2min, and plate read at 83 ℃ for 10s (35cyles) | (M. and E., 1999) |
| Archaeal *amoA* | Arch-amoAF  Arch-amoAR | STAATGGTCTGGCTTCTTC  GCGGCATCCATCTGTCTGT | 635 | 95°C for 3 min; 35 circles of (95°C for 10s, 55°C for 30s, 72°C for 60s and plate read at 83°C for 10s) | (CA et al., 2005) |
| Bacterial *amoA* | amoA-1F  amoA-2R | GGGGTTTCTACTGGTGGT  CCCCTCKGSAAAGCCTTCTTC | 490 | 95°C for 3 min; 35 circles of (95°C for 10s, 55°C for 30s, 72°C for 60s and plate read at 83°C for 10s) | (Zeng et al., 2011) |
| *nirK* | F1aCu  R3Cu | ATCATGGTSCTGCCGCG  GCCTCGATCAGRTTGTGGTT | 476 | 95°C for 5min; 95℃ for 30s, 58℃ (a touchdown from 63℃ to 58℃, -1.0℃,6 circles) for 30s, 72℃ for 1min and plate read at 83 ℃ for 10s (40cyles) | (Zhang et al., 2019) |
| *nirS* | cd3aF  R3cd | GTSAACGTSAAGGARACSGG  GASTTCGGRTGSGTCTTGA | 420 | 94°C for 2min; 94℃ for 30s, 53℃ (a touchdown from 58℃ to 53℃, -1.0℃,5 circles) for 1min, 72℃ for 30s and plate read at 83 ℃ for 10s (30cyles) | (Liu et al., 2022) |
| *nosZ* | nosZ_2R  nosZ_2F | CGCRACGGCAASAAGGTSMSSGT  CAKRTGCAKSGCRTGGCAGAA | 453 | 95°C for 10min; 95℃ for 30s, 60℃ (a touchdown from 65℃ to 60℃, -1.0℃, 5 circles) for 30s, 72℃ for 1min and plate read at 83 ℃ for 10s (40cyles) | (Henry et al., 2006) |
| *nosZ*(Clade II) | nosZ- II-F  nosZ-II-R | CTIGGICCIYTKCAYAC  GCIGARCARAAITCBGTRC | 698 | 95°C for 2min; 95℃ for 30s, 54℃ for 30s, 72℃ for 40s and plate read at 83 ℃ for 10s (40cyles) | (Liu et al., 2022) |

**Table S3** The *α* diversity of *pmoA*

| Treatment | Chao | Coverage | Observed-species | Shannon | Simpson | Pielou-e |
| --- | --- | --- | --- | --- | --- | --- |
| R-CK | 2475.49±159.07c | 0.967±0.002a | 1203.30±43.37cd | 5.34±0.09a | 0.910±0.012a | 0.5215±0.0059a |
| R-B300-M | 2459.62±213.76c | 0.968±0.003a | 1125.00±150.53bcd | 5.10±0.78a | 0.888±0.056a | 0.5027±0.0673a |
| R-B300-H | 2487.39±337.80c | 0.966±0.005a | 1319.27±227.59d | 5.50±0.62a | 0.907±0.029a | 0.5308±0.0483a |
| R-B500-M | 2340.90±146.20c | 0.969±0.002ab | 1103.50±46.96abcd | 5.07±0.05a | 0.896±0.006a | 0.5015±0.0016a |
| R-B500-H | 2097.60±460.53bc | 0.971±0.008abc | 1145.93±353.99bcd | 5.20±0.80a | 0.881±0.045a | 0.5130±0.0567a |
| B-CK | 1429.17±320.90a | 0.982±0.004d | 737.70±155.60a | 4.71±0.24a | 0.891±0.014a | 0.4966±0.0097a |
| B-B300-M | 1752.07±142.67ab | 0.976±0.003bcd | 937.57±139.55abc | 4.82±0.11a | 0.892±0.012a | 0.4887±0.0135a |
| B-B300-H | 1688.79±87.57ab | 0.978±0.001cd | 776.10±49.79ab | 4.80±0.19a | 0.907±0.016a | 0.5005±0.0160a |
| B-B500-M | 1434.94±107.89a | 0.981±0.002d | 824.73±36.39abc | 5.14±0.01a | 0.908±0.002a | 0.5304±0.0047a |
| B-B500-H | 1541.90±128.57a | 0.979±0.002d | 835.43±81.52abc | 5.26±0.45a | 0.926±0.020a | 0.5423±0.0393a |

Data are represented by their means and standard deviation (n = 3). Lowercase letters indicate significant differences among different treatments (*P*<0.05).

**Table S4** The *α* diversity of *nosZ*

| Treatment | Chao | Coverage | Observed species | Shannon | Simpson | Pielou-e |
| --- | --- | --- | --- | --- | --- | --- |
| R-CK | 4313.21±746.46d | 0.9871±0.0026a | 3282.30±552.02c | 8.08±0.21ab | 0.9879±0.0014a | 0.693±0.010a |
| R-B300-M | 4041.59±314.65cd | 0.9884±0.0008ab | 3132.00±241.36bc | 8.10±0.42ab | 0.9860±0.0045a | 0.697±0.030a |
| R-B300-H | 2961.55±426.53ab | 0.99280.0017cd | 2429.17±453.03ab | 7.70±0.55ab | 0.9809±0.0077a | 0.686±0.034a |
| R-B500-M | 4085.76±167.78cd | 0.9882±0.0007ab | 3134.37±108.29bc | 7.87±0.20ab | 0.9795±0.0109a | 0.678±0.018a |
| R-B500-H | 3114.29±194.67abc | 0.9912±0.0009bcd | 2371.77±152.43ab | 7.27±0.32a | 0.9737±0.0082a | 0.648±0.032a |
| B-CK | 3873.30±558.37bcd | 0.9896±0.0019abc | 3106.50±398.54bc | 8.25±0.21b | 0.9887±0.0008a | 0.712±0.008a |
| B-B300-M | 3340.69±542.11abcd | 0.9911±0.0013bcd | 2717.10±485.35abc | 7.52±0.88ab | 0.9615±0.0370a | 0.659±0.064a |
| B-B300-H | 2517.46±235.60a | 0.9932±0.0007d | 1930.07±203.96a | 7.49±0.26ab | 0.9834±0.0047a | 0.687±0.020a |
| B-B500-M | 3894.45±563.20bcd | 0.9891±0.0018ab | 3055.13±433.63bc | 8.04±0.21ab | 0.9868±0.0010a | 0.696±0.006a |
| B-B500-H | 3841.71±86.93bcd | 0.9896±0.0004abc | 3095.73±36.47bc | 8.22±0.17ab | 0.9877±0.0021a | 0.709±0.014a |

Data are represented by their means and standard deviation (n = 3). Lowercase letters indicate significant differences among different treatments (*P*<0.05).

# Reference

Ba, H., C, E., Ritchie Da, Hall G, Pickup Rw, and Jr, S. (1996). Isolation and identification of methanogen-specific DNA from blanket bog feat by PCR amplification and sequence analysis. *Appl. Environ. Microbiol.* 62**,** 668–675.doi:10.1128/aem.62.2.668-675.1996

Ca, F., Kj, R., Jm, B., Ae, S., and Bb, O. (2005). Ubiquity and diversity of ammonia-oxidizing Archaea in water columns and sediments of the ocean. *PNAS.* 102**,** 14683–14688.doi:10.1073pnas.0506625102

Henry, S., Bru, D., Stres, B., Hallet, S., and Philippot, L. (2006). Quantitative detection of the *nosZ* gene, encoding nitrous oxide reductase, and comparison of the abundances of 16S rRNA, *narG*, *nirK*, and *nosZ* genes in soils. *Appl. Environ. Microbiol.* 72**,** 5181-5189.doi:10.1128/AEM.00231-06

Liu, H., Zheng, X., Li, Y., Yu, J., Ding, H., Sveen, T.R., et al. (2022). Soil moisture determines nitrous oxide emission and uptake. *Sci. Total Environ.* 822**,** 153566.doi:10.1016/j.scitotenv.2022.153566

M., C.A., and E., L.M. (1999). Molecular characterization of functional and phylogenetic genes from natural populations of methanotrophs in lake sediments. *Appl. Environ. Microbiol.* 65**,** 5066-5074.doi:10.1128/AEM.65.11.5066-5074.1999

Zeng, G., Zhang, J., Chen, Y., Yu, Z., Yu, M., Li, H., et al. (2011). Relative contributions of archaea and bacteria to microbial ammonia oxidation differ under different conditions during agricultural waste composting. *Bioresour. Technol.* 102**,** 9026-9032.doi:10.1016/j.biortech.2011.07.076

Zhang, Song, Zhou, Cao, and Zhou. (2019). Coupling between Nitrification and Denitrification as well as Its Effect on Phosphorus Release in Sediments of Chinese Shallow Lakes. *Water* 11,1809.doi:10.3390/w11091809
